# Data-Driven Identification of Factors That Influence the Quality of Adverse Event Reports: 15-Year Interpretable Machine Learning and Time-Series Analyses of VigiBase and QUEST

**DOI:** 10.2196/49643

**Published:** 2024-04-03

**Authors:** Sim Mei Choo, Daniele Sartori, Sing Chet Lee, Hsuan-Chia Yang, Shabbir Syed-Abdul

**Affiliations:** 1 Centre of Compliance & Quality Control National Pharmaceutical Regulatory Agency Petaling Jaya Malaysia; 2 Graduate Institute of Biomedical Informatics Taipei Medical University Taipei Taiwan; 3 Uppsala Monitoring Centre Uppsala Sweden; 4 International Center for Health Information Technology Taipei Medical University Taipei Taiwan; 5 Clinical Big Data Research Center Taipei Medical University Hospital Taipei Medical University Taipei Taiwan; 6 Research Center of Big Data and Meta-Analysis Wan Fang Hospital Taipei Medical University Taipei Taiwan; 7 School of Gerontology and Long-Term Care Taipei Medical University Taipei Taiwan

**Keywords:** pharmacovigilance, medication safety, big data analysis, feature selection, interpretable machine learning

## Abstract

**Background:**

The completeness of adverse event (AE) reports, crucial for assessing putative causal relationships, is measured using the vigiGrade completeness score in VigiBase, the World Health Organization global database of reported potential AEs. Malaysian reports have surpassed the global average score (approximately 0.44), achieving a 5-year average of 0.79 (SD 0.23) as of 2019 and approaching the benchmark for well-documented reports (0.80). However, the contributing factors to this relatively high report completeness score remain unexplored.

**Objective:**

This study aims to explore the main drivers influencing the completeness of Malaysian AE reports in VigiBase over a 15-year period using vigiGrade. A secondary objective was to understand the strategic measures taken by the Malaysian authorities leading to enhanced report completeness across different time frames.

**Methods:**

We analyzed 132,738 Malaysian reports (2005-2019) recorded in VigiBase up to February 2021 split into historical International Drug Information System (INTDIS; n=63,943, 48.17% in 2005-2016) and newer E2B (n=68,795, 51.83% in 2015-2019) format subsets. For machine learning analyses, we performed a 2-stage feature selection followed by a random forest classifier to identify the top features predicting well-documented reports. We subsequently applied tree Shapley additive explanations to examine the magnitude, prevalence, and direction of feature effects. In addition, we conducted time-series analyses to evaluate chronological trends and potential influences of key interventions on reporting quality.

**Results:**

Among the analyzed reports, 42.84% (56,877/132,738) were well documented, with an increase of 65.37% (53,929/82,497) since 2015. Over two-thirds (46,186/68,795, 67.14%) of the Malaysian E2B reports were well documented compared to INTDIS reports at 16.72% (10,691/63,943). For INTDIS reports, higher pharmacovigilance center staffing was the primary feature positively associated with being well documented. In recent E2B reports, the top positive features included reaction abated upon drug dechallenge, reaction onset or drug use duration of <1 week, dosing interval of <1 day, reports from public specialist hospitals, reports by pharmacists, and reaction duration between 1 and 6 days. In contrast, reports from product registration holders and other health care professionals and reactions involving product substitution issues negatively affected the quality of E2B reports. Multifaceted strategies and interventions comprising policy changes, continuity of education, and human resource development laid the groundwork for AE reporting in Malaysia, whereas advancements in technological infrastructure, pharmacovigilance databases, and reporting tools concurred with increases in both the quantity and quality of AE reports.

**Conclusions:**

Through interpretable machine learning and time-series analyses, this study identified key features that positively or negatively influence the completeness of Malaysian AE reports and unveiled how Malaysia has developed its pharmacovigilance capacity via multifaceted strategies and interventions. These findings will guide future work in enhancing pharmacovigilance and public health.

## Introduction

### Background

Pharmacovigilance (PV) is the science and activities related to the detection, assessment, understanding, and prevention of adverse effects or any other possible drug-related problems [1]. Individual case safety reports (ICSRs) of suspected adverse drug reactions and adverse events following immunization (hereafter collectively referred to as adverse events [AEs]) collected in spontaneous reporting systems (SRSs) remain the cornerstone of postmarketing drug safety surveillance [2,3] (see Multimedia Appendix 1 for a list of definitions [4-10]).

Over 170 participating countries in the World Health Organization (WHO) Programme for International Drug Monitoring (PIDM) share reports of suspected AEs and collaborate worldwide in monitoring and identifying signals of AEs [4]. The WHO PIDM signal detection process is anchored on data recorded in the WHO global ICSR database, VigiBase, developed and maintained by the WHO Collaborating Centre for International Drug Monitoring, Uppsala Monitoring Centre (UMC), Sweden. Common technical specifications for report transmission and standard terminologies for drugs and reactions have evolved over the years to facilitate global information sharing and efficient analysis [4,5]. Currently, VigiBase accepts 3 standard formats: the original International Drug Information System (INTDIS) and 2 revisions of the International Council for Harmonisation of Technical Requirements for Pharmaceuticals for Human Use (ICH) Guidelines for the Electronic Transmission of ICSRs, namely E2B(R2), and the latest E2B(R3), with all data being transformed to a format most closely resembling E2B(R2) in VigiBase [4].

The participating members are characterized by diverse contexts—sociocultural, political, and clinical—that affect the measures in which reports are collected and processed, as well as their quality [4]. Ideally, a robust PV system should consider all data quality parameters, including accuracy, completeness, conformity, consistency, currency, duplication, integrity, precision, relevance, and understandability. Among all these parameters, problems associated with completeness (ie, missing data) have long been regarded as critical factors hampering the usefulness of existing reports [5,6]. Influxes of poorly documented reports could increase operational burdens, upsurge a system’s resources, and even mask or delay the detection of drug safety signals [11].

While only 4 elements (identifiable patient, identifiable reporter, medicinal product, and AE) are required for a valid report, they are often insufficient for productive analyses of potential causal relationships between medicinal products and AEs [6]. In 2014, the UMC developed the vigiGrade completeness score, an automated multidimensional tool that measures the amount of clinically relevant information in reports essential for causality assessment, replacing the 4-grade WHO documentation grading scheme since the 1990s [6,12]. The vigiGrade score quantifies report completeness based on a selection of ICH-E2B fields: time to onset, indication, event outcome, patient age and sex, dose information, country of origin, reporter, type of report, and free-text fields. The vigiGrade score can be used to pinpoint trends in report quality over time and reflect systematic data quality issues in collections of reports from member countries. For instance, vigiGrade uncovered miscoded age units in US reports and missing AE outcomes in Italian reports [6]. The score may also guide reviewers in judging whether the information in a report suffices for a problem to be investigated [4]. Notably, vigiGrade has proven to be an indicator of a true signal and is part of the data-driven predictive model used by the UMC, vigiRank, for signal detection [13].

### The PV System in Malaysia

PV activities in Malaysia began in the 1980s with the establishment of the Malaysian Adverse Drug Reactions Advisory Committee (MADRAC) under the Drug Control Authority (DCA) [7]. The Malaysian national PV center is based within the National Pharmaceutical Regulatory Agency (NPRA) under the Pharmaceutical Services Programme of the Ministry of Health (MOH). Malaysia became a member of the WHO PIDM in 1990 and is regarded as an established PV center, receiving >30,000 reports annually, which is well above the WHO criteria of 200 reports per million inhabitants per year since 2009. Every AE report recorded in the national PV database (QUEST; see Multimedia Appendix 1 [7] for a detailed description) is carefully processed and assessed by trained pharmacists at the national center and subsequently reviewed by the MADRAC before submission to the UMC for inclusion in VigiBase (Figure S1 in Multimedia Appendix 2).

### Problem Statement and Research Benefits

Previous studies have not evaluated the quality of Malaysian AE reports, and little is known about the underlying factors affecting their vigiGrade completeness scores. However, identifying and validating factors associated with report quality was made difficult by the large number and variety of potentially correlated characteristics of a spontaneous report—at the reaction, drug, patient, reporter, sender, or regulator level (see the literature review on AE report quality in Multimedia Appendix 3 [6,14-38]). As of 2019, Malaysian reports demonstrated a 5-year average completeness score of 0.79, surpassing the global average of approximately 0.44 in VigiBase and approaching the benchmark for well-documented reports (0.80). Therefore, this study primarily aimed to use a hypothesis-free, data-driven approach to explore the main drivers influencing the completeness of Malaysian reports in VigiBase over a 15-year period using vigiGrade. A secondary objective was to understand the strategic measures taken by the Malaysian authorities that preceded the relatively high completeness score across different time frames. A better understanding of the drivers of AE report completeness may be helpful for the NPRA and regulators worldwide.

## Methods

### Data-Driven Framework for Identifying Factors Associated With AE Report Quality

#### Overview

Our study used big data analysis approaches incorporating machine learning (ML) methods, which are becoming increasingly prevalent in clinical and epidemiological research [8,39]. These approaches aimed to overcome limitations inherent in traditional approaches in handling complex interactions among variables (eg, multicollinearity and nonlinearity). Importantly, ML methods focus on identifying patterns and associations within complex data rather than on establishing causal inference [39]. For clarity, we have outlined the similarities in concepts and nomenclatures between ML and traditional medical statistics in Multimedia Appendix 1.

#### Hybrid Feature Selection

Our study leveraged ML methods for feature selection, mitigating human bias in analyzing extensive report characteristics, which might be overlooked by traditional hypothesis-driven approaches that are prone to high selection biases [9]. We combined statistical filtering and ML algorithms to preselect features, reducing overfitting risks (often arising from redundancy and multicollinearity) and computational costs [40,41]. Notably, Stevens et al [9] used random forest (RF)–based feature selection to identify potential risk factors associated with cardiovascular diseases. In almost all domains, incorporating domain expertise remains vital for developing meaningful and effective models [8]. Multimedia Appendix 4 [8,9,39-48] provides detailed explanations.

#### Interpretable ML

Post hoc explanation methods such as Shapley additive explanations (SHAP) have been increasingly used to provide interpretability for complex black-box models such as RF [49]. Van den Bosch et al [50] used regression coefficients and SHAP value analyses to identify risk factors associated with 30-day mortality among patients undergoing colorectal cancer surgery. Gong et al [51] also developed an ML framework for acute kidney injury prediction and interpretation using SHAP values to assess feature contributions and identify specific patient impacts. Multimedia Appendix 5 [49-57] provides detailed explanations.

### Study Design

**Figure 1:**
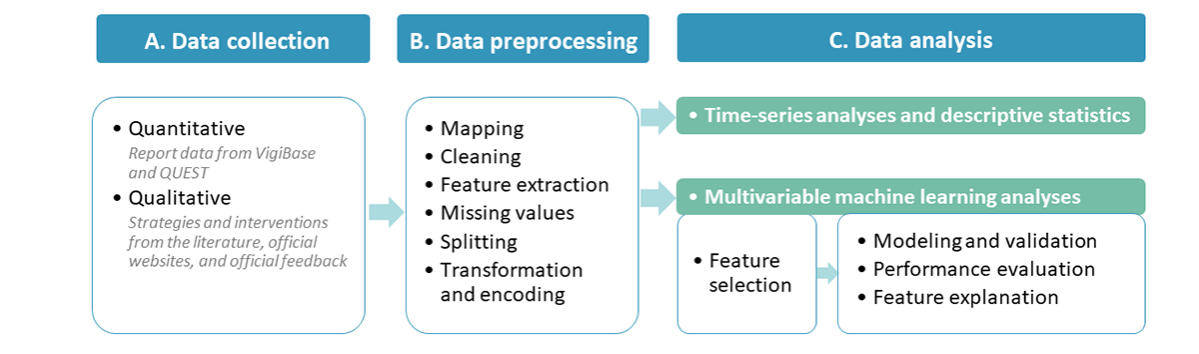
Overview of study workflow.

This observational study used interpretable ML and descriptive time-series analyses of PV database data. Fundamentally, it was an exploratory data analysis assessing a large number of report characteristics without prespecified hypotheses [42] aiming to identify factors influencing report quality. The main steps of our methodological workflow are illustrated in Figure 1.

### Data Collection

AE report data were obtained from VigiBase in CSV format. Supplementary information, such as means of reporting, sender type, and sender region, was retrieved from the NPRA QUEST3+ (latest version) database in CSV format. For the secondary objective of understanding the key drivers of AE report completeness, strategies and interventions implemented in Malaysia were collected from the literature, official websites, and feedback from the NPRA. We included all reports recorded in VigiBase as of February 2021; reported in Malaysia; and received by the NPRA from January 1, 2005, to December 31, 2019. This 15-year range was chosen for its relevance to current PV needs and to enable the timely identification of necessary improvements. We excluded reports that (1) were suspected duplicates identified by the UMC’s vigiMatch [4] (see Multimedia Appendix 1 for operational definitions), (2) were not sourced from Malaysia, (3) had null average completeness scores, and (4) lacked drugs marked as suspected or interacting.

### Study Variables

#### Dependent Variables or Outcomes

The vigiGrade completeness score (*C*; ranges from 0.07 to 1) was classified as well documented (*C*>0.8) or not well documented (*C*≤0.8; see Multimedia Appendix 1 for operational definitions).

#### Independent Variables or Explanatory Features

The variables related to administrative, sender, reporter, patient, drug, and reaction characteristics are presented in Table S1 in Multimedia Appendix 2.

### Data Preprocessing

#### Data Mapping

Supplementary data from QUEST3+ were mapped to the primary data set from VigiBase using the primary identifier. Given the distinct differences in reporting elements of INTDIS and E2B formats (input values vary in certain data fields), we divided the data set for separate analysis.

#### Data Cleaning and Feature Extraction

We cleaned and engineered the features from the available data based on the literature, domain knowledge, and previous experience. Information about the WHO Anatomical Therapeutic Chemical (ATC) classification system codes was provided by the UMC in the data set. If an active ingredient was linked to more than one code, an ATC level-2 code was manually assigned based on indication, route of administration, dosage, product information, and clinical narratives. Reporting qualifications in INTDIS format were harmonized with the E2B format with reference to supplementary data from the NPRA. We calculated the number of suspected or interacting drugs, concomitant drugs, and reactions for each report. We also included the annual staffing level of the national PV center in Malaysia and the means of reporting (based on report identifier).

#### Missing Values

Continuous variables consisting of null values were converted to categorical variables based on data distribution and domain knowledge. Missing values for categorical variables were grouped as a *null* category.

#### Data Splitting

To ensure a consistent distribution of target classes, we applied stratified random sampling. We allocated 90% of the data for training, which underwent 10-fold cross-validation, and reserved 10% for testing to gauge model performance on unseen samples. Our approach prioritized the extraction of insights from the current data set rather than overgeneralizations on future data.

#### Transformation and Encoding

To overcome data complexity and maximize interpretability, data at the drug event level were transformed to the case (report) level. Observations related to concomitant drugs were excluded as the vigiGrade scoring method is restricted to drugs listed as suspected or interacting [6]. We took the average value of a case for continuous variables whereby, for categorical variables, we examined the presence (or absence) of a particular drug- or event-related characteristic. Continuous variables were standardized. Binary categorical variables such as patient sex were integer encoded. One-hot encoding was performed on the remaining categorical variables, including ordinal variables and categories labelled as *null* or *unknown*. In the following sections, we distinguish variables from features, where the latter correspond to the processed variables in a binary fashion for the ML model input [43,56].

### Multivariable ML Analysis

#### Feature Selection

We performed hybrid feature selection to eliminate redundant or less informative features before data mining using the ML algorithm. To avoid data leakage and the corresponding model overfitting, we conducted a 2-stage feature selection solely based on training data [8,44]. We first applied the univariable filter method to independently assess and preselect the features and subsequently selected the top-ranked features using RF-based recursive feature elimination coupled with multicollinearity assessment. The detailed processes are provided in Multimedia Appendix 4.

#### Modeling and Validation

We applied a supervised ML method to identify key features relevant to the reports classified as well documented. Specifically, the RF classifier was selected for its robustness to nonparametric distributions, nonlinearity, and outliers [58] and its out-of-the-box performance. Its built-in feature importance metrics allowed us to assess the relative attribution of a feature to the classification task. The more a feature is used to make key decisions with the forest of decision trees, the higher its relative importance. To mitigate class imbalance in the INTDIS data set, we used RandomUnderSampler with a 0.25 ratio that achieved optimal balanced performance of prediction and recall. We chose undersampling over synthetic sampling methods to preserve the real-world data characteristics. For the imbalanced INTDIS data set, we adjusted the class_weight parameter in the RF classifier to *balanced*. We evaluated the RF classification models using 10-fold cross-validation.

#### Performance Evaluations

Classification performance was measured using the area under the receiver operating characteristic curve, accuracy, recall (sensitivity), and precision (positive predictive values). For the imbalanced INTDIS data set, *F*_1_-scores (harmonic average of precision and recall) were reported.

#### Feature Explanations

To mitigate the issue of black-box predictions**,** we used TreeExplainer [52] to generate SHAP summary plots that succinctly display the magnitude, prevalence, and direction of a feature’s effect by measuring each feature’s attributions to the classification. In SHAP, the feature effect is a measure of how much the value of a specific feature influences the prediction made by the model.

#### Software and Packages

All ML analyses were developed in Jupyter Notebook (Project Jupyter) using Python (version 3.7.9; Python Software Foundation). Statistical tests were performed using *pandas* (version 1.2.4) and *statsmodels* (version 0.12.2) [59]. ML analysis was completed using the *scikit-learn* package (version 0.24.2) [60]. SHAP values were calculated using TreeExplainer [52].

### Time-Series and Descriptive Statistical Analysis

We used time-series analysis and descriptive statistics to evaluate the trends in report quality and the characteristics associated with well-documented reports over different time frames. One-way ANOVA or 2-tailed Student *t* tests were conducted on continuous variables, whereas the chi-square or Fisher exact test was used to compare categorical variables, as appropriate. A *P* value of <.05 was considered statistically significant. All analyses were conducted using the SAS software (version 9.4; SAS Institute).

### Ethical Considerations

This study was registered with the Malaysian National Medical Research Register (NMRR-20-983-53984 [Investigator Initiated Research]) and received ethics approval from the Medical Review and Ethics Committee, MOH, Malaysia (reference: KKM/NIHSEC/P20–1144(4)).

## Results

### Overview

We analyzed the completeness of Malaysian AE reports in VigiBase received by the NPRA over 15 years. A total of 132,738 reports were included in the analysis following the predefined inclusion and exclusion criteria (Figure S2 in Multimedia Appendix 2). Table S1 in Multimedia Appendix 2 summarizes the characteristics of the INTDIS and E2B reports included in this study concerning administration, reporter, patient, drug, and reaction by status of being well documented. Among the included reports, 48.17% (63,943/132,738) were in the INTDIS format, and 51.83% (68,795/132,738) were in the E2B format. Over two-thirds (46,186/68,795, 67.14%) of E2B reports were well documented compared to 16.72% (10,691/63,943) of INTDIS reports.

### Multivariable ML Analysis

#### Selected Features

For the INTDIS subsets, 90 features were preselected using univariate filter methods and further narrowed down to 33 features following RF-based recursive feature elimination ranking and multicollinearity assessment. For the E2B subsets***,*** 90 features were preselected and subsequently reduced to 40.

#### Classification Performance

The performance of the RF models in classifying reports as well or not well documented is presented in Table 1.

**Table 1:** Classification performance of random forest model for the training (10-fold cross-validation) and test set.

			Recall (%)		Precision (%)		Accuracy (%)		AUROC^a^ (%)		*F*_1_-score (%)
**INTDIS^b^**											
	Training, mean (SD)	99.6 (0.03)		95.4 (0.13)		99.0 (0.03)		99.8 (0.01)		97.4 (0.06)	
	Validation, mean (SD)	73.7 (1.90)		77.0 (1.02)		90.3 (0.39)		95.0 (0.33)		75.3 (1.16)	
	Test	74.9		74.3		91.5		95.1		74.6	
**E2B**											
	Training, mean (SD)	99.7 (0.02)		99.7 (0.02)		99.6 (0.01)		99.9 (0.001)		99.7 (0.01)	
	Validation, mean (SD)	96.9 (0.27)		91.6 (0.34)		92.0 (0.24)		94.9 (0.29)		94.2 (0.20)	
	Test	96.9		90.9		91.4		95.1		93.8	

^a^AUROC: area under the receiver operating characteristic curve.

^b^INTDIS: International Drug Information System.

#### Top Factors Predicting Status of Malaysian Reports Being Well Documented

##### RF ML Model

Figure S3 in Multimedia Appendix 2 reveals the top-ranked features that contributed to the status of INTDIS and E2B reports being well documented derived from the RF ML model’s built-in feature importance metrics. However, the directions of their contribution were not known due to the black-box nature of the RF model. For INTDIS reports received between 2005 and 2016, PV center staffing was identified as the most important factor in predicting their status of being well documented. Other important factors included suspected drug withdrawal, the number of reactions reported, reaction abated upon drug dechallenge, and patient sex. Reaction abated upon drug dechallenge, on the other hand, appeared to be the most important factor predicting whether an E2B report received between 2015 and 2019 was well documented. Reports from other health care professionals (HCPs), reactions occurring <1 day, reports submitted by product registration holders (PRHs), and the number of concomitant drugs were also among the top 5 important factors.

##### SHAP Interpretation Method

**Figure 2:**
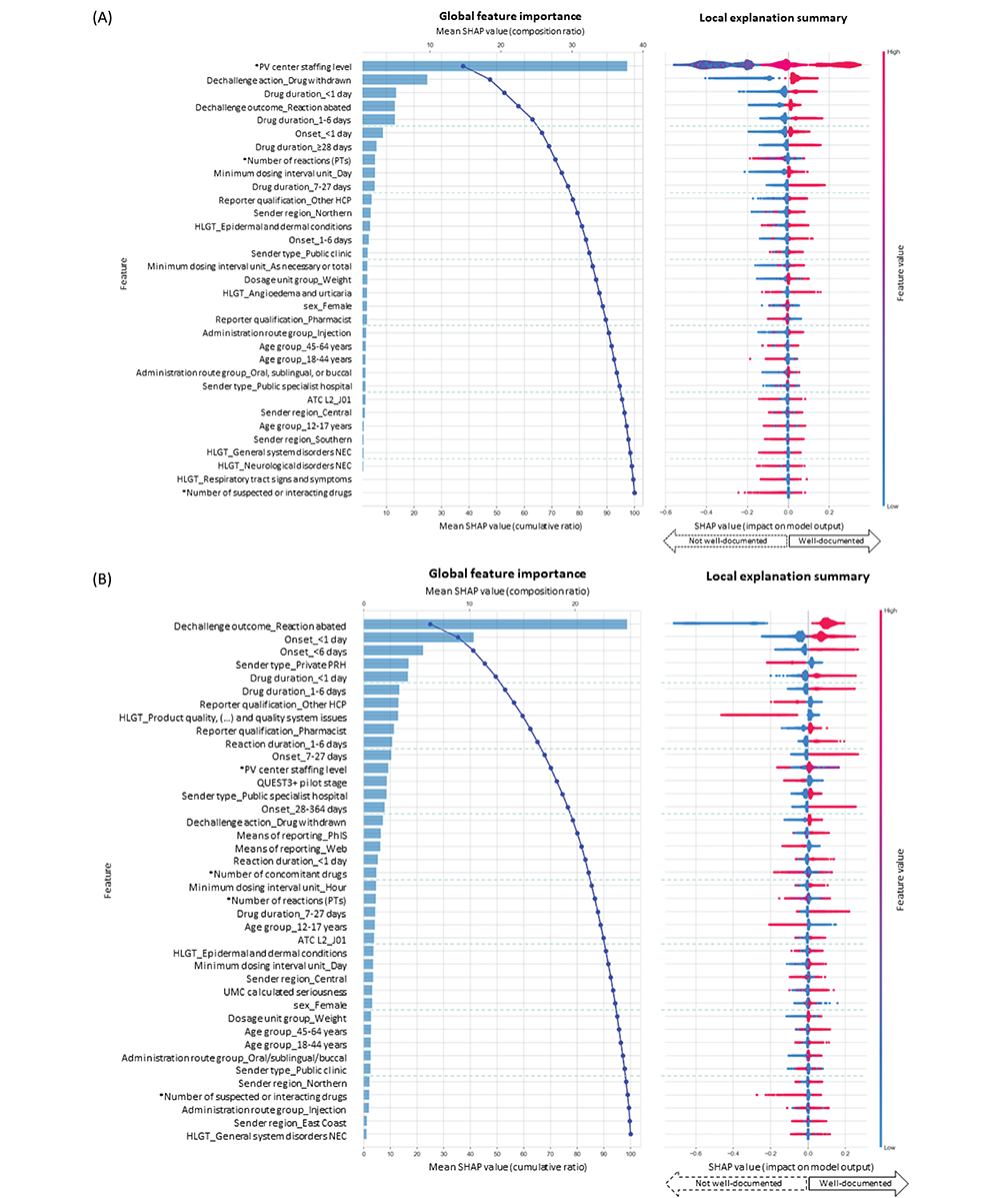
(A) Top 33 features for the International Drug Information System (INTDIS) subset from 2005 to 2016; (B) top 40 features for the E2B subset during the years 2015 to 2019. The Shapley additive explanation (SHAP) bar plot illustrates global feature importances based on mean absolute SHAP values, highlighting the impact of each feature on the model’s predictions. Higher values represent greater influence. The waterfall plot indicates the cumulative contribution of features to the model. The SHAP summary plot of local explanations displays each observation as a dot, with its position on the x-axis (SHAP value) indicating the impact of a feature on the model’s classification for that observation. Continuous features (marked with an asterisk) range from low (blue) to high (red) values, whereas categorical features of a binary nature are either absent (blue) or present (red). The distribution of dots indicates the magnitude and prevalence of a feature effect. Features are ordered by global importance. ATC: Anatomical Therapeutic Chemical; HCP: health care professional; HLGT: Medical Dictionary for Regulatory Activities High-Level Group Term; NEC: not elsewhere classified; PhIS: pharmacy hospital information system; PRH: product registration holder; PT: Medical Dictionary for Regulatory Activities Preferred Term; PV: pharmacovigilance; UMC: Uppsala Monitoring Centre; UMC calculated seriousness: serious cases classified automatically by a UMC-developed algorithm.

The SHAP post hoc interpretations provided us with an understanding of the magnitude, prevalence, and direction of feature effects. Figure 2 depicts rich summaries of individual attributions for all features, allowing us to discover key factors that influence well-documented reports. Features with higher global importance have a greater influence on the model’s predictions. A feature with predominantly red dots to the right (eg, reaction abated upon drug dechallenge in Figure 2B) implies a positive contribution to well-documented reports, whereas a negative contribution is indicated if the direction is to the left (eg, reports submitted by PRHs in Figure 2B). Features with lower global importance but a long tail stretching in one direction indicate a rare but high-magnitude effect [52,56]. As mean SHAP values are calculated across all cases, a feature with a lower impact but higher prevalence may have a higher SHAP value [50]. For the least globally important features, we observed that their feature effects were not constant across cases, with blue and red dots dispersed in both directions. This variation may arise from interactions with other features that modulate their importance in different cases [52].

Regarding INTDIS reports, in earlier years, PV center staffing was the primary factor driving the Malaysian rate of reports being well documented, with this factor alone accounting for >35% of the model’s explainability. The next most important factor favoring well-documented reports was drug withdrawal, followed by a duration of drug use of <1 week, reaction abated upon drug dechallenge, and reaction occurring <1 day. In contrast, an increased number of reported reactions and reports from pharmacists predicted not well-documented INTDIS reports.

In more recent years, the most important factor favoring well-documented E2B reports from Malaysia was reaction abated upon drug dechallenge, which alone was responsible for >25% of the model’s explainability. Among the top 25 features, which provided 90% of the model’s interpretation on classifying status of being well documented, 6 (24%) were found to be negatively associated with well-documented reports: reports submitted by PRHs; reports made by other HCPs; reactions under the Medical Dictionary for Regulatory Activities (MedDRA) High-Level Group Terms (HLGTs) *product quality, supply, distribution, manufacturing,* and *quality system issues*; reports received during the QUEST3+ pilot stage, reports received via web reporting, and adolescent patients (aged 12-17 years). E2B reports that involved reactions with a shorter time to onset and duration of drug use were more likely to be well documented. Other identified key drivers of Malaysian well-documented E2B reports were reports made by pharmacists, reports submitted from public specialist hospitals, pharmacy hospital information system (PhIS)–integrated reporting, and the involvement of systemic antimicrobials (ATC code J01).

### Time-Series and Descriptive Statistical Analysis

#### Trend Analysis of Malaysian AE Report Quality (2005-2019)

**Figure 3:**
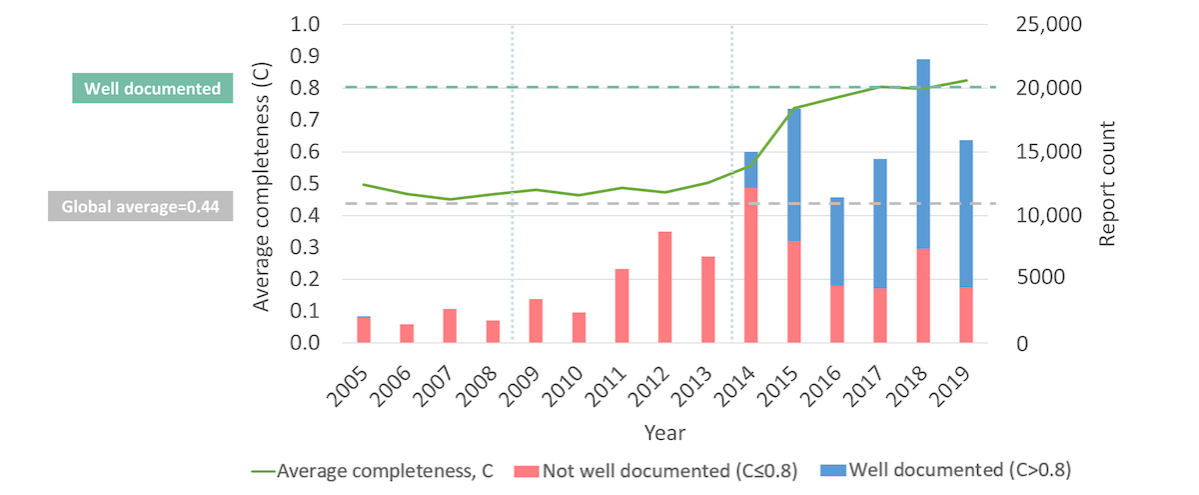
Distribution of average completeness scores and counts of Malaysian reports by status of being well documented in VigiBase over the study period.

Figure 3 depicts the time trends in AE reporting in Malaysia, illustrating how both the quantity and completeness scores of AE reports received by the NPRA grew over a 15-year period from 2005 to 2019. In Tables 2 and 3, we summarize the trends divided into 5-year subperiods, further stratified by sender type and reporter qualification. Of the total 132,738 reports received, 56,877 (42.84%) were well documented. Before 2014, the average completeness score consistently fell short of 0.5 but was slightly above the global average of 0.44. The volume of reports surged by 121% from 2013 to 2014, whereas the proportion of well-documented reports rose from practically 0% to 18.93% (2843/15,013). Since 2015, more than half (53,929/82,497, 65.37%) of the Malaysian reports were well documented, averaging 0.79 (SD 0.23) over the last 5 years, with a new high of 0.82 in 2019.

**Table 2:** The 5-year stratified summary statistics of overall Malaysian report quality.

	Total	2005-2009	2010-2014	2015-2019	*P* value^a^
Overall reports, n (%)	132,738 (100)	11,458 (8.6)	38,783 (29.2)	82,497 (62.2)	N/A^b^
Completeness, mean (SD)	0.68 (0.25)	0.47 (0.15)	0.51 (0.18)	0.79 (0.23)	<.001
Well-documented reports (*C*^c^>0.8), n (%)	56,877 (42.8)	105 (0.9)	2843 (7.3)	53,929 (65.4)	<.001

^a^*P* value based on ANOVA.

^b^N/A: not applicable.

^c^vigiGrade completeness score.

**Table 3:** The 5-year stratified summary statistics of well-documented Malaysian reports (N=56,877).

		Total, n (%)	2005-2009 (n=105), n (%)	2010-2014 (n=2843), n (%)	2015-2019 (n=53,929), n (%)	*P* value^a^	
**Sender type**							<.001
	Public specialist hospital	31,503 (55.4)	72 (68.6)	1542 (54.2)	29,889 (55.4)		
	Public nonspecialist hospital	4954 (8.7)	3 (2.9)	217 (7.6)	4734 (8.8)		
	Public clinic	15,609 (27.4)	4 (3.8)	866 (30.5)	14,739 (27.3)		
	Other public services	3 (0)	0 (0)	1 (0)	2 (0)		
	University hospital	496 (0.9)	9 (8.6)	25 (0.9)	462 (0.9)		
	Private PRH^b^	939 (1.7)	11 (10.5)	58 (2)	870 (1.6)		
	Private hospital or clinic	2114 (3.7)	6 (5.7)	106 (3.7)	2002 (3.7)		
	Private community pharmacy	45 (0.1)	0 (0)	0 (0)	45 (0.1)		
	Consumer	30 (0.1)	0 (0)	0 (0)	30 (0.1)		
	Unknown	1184 (2.1)	0 (0)	28 (1)	1156 (2.1)		
**Reporter qualification**							<.001
	Physician	11,446 (20.1)	53 (50.5)	488 (17.2)	10,905 (20.2)		
	Pharmacist	40,295 (70.8)	16 (15.2)	1850 (65.1)	38,429 (71.3)		
	Other HCP^c^	4084 (7.2)	36 (34.3)	500 (17.6)	3548 (6.6)		
	Consumer	78 (0.1)	0 (0)	0 (0)	78 (0.1)		
	Unknown	974 (1.7)	0 (0)	5 (0.2)	969 (1.8)		

^a^*P* value based on the Fisher exact test.

^b^PRH: product registration holder.

^c^HCP: health care professional.

Over the 15 years, most well-documented reports in Malaysia came from public health facilities, with public specialist hospitals contributing more than half (31,503/56,877, 55.38%). Public clinics emerged as key contributors in later stages, with well-documented reports increasing considerably from 3.8% (4/105) in the period from 2005 to 2009 to 30.46% (866/2843) in the following 5 years. Compared to public services, the private sector consistently demonstrated a marginal contribution to quality AE reporting in Malaysia. In the earlier years, physicians contributed approximately half (53/105, 50.5%) of the well-documented reports. In the subsequent periods, reports from pharmacists showed a rise in quantity and average completeness, yielding the highest overall rate of being well documented (1850/2843, 65.07% to 38,429/53,929, 71.26%) among all reporter types from 2010 to 2019.

#### Key Strategies and Interventions Implemented in Malaysia (2005-2019)

**Figure 4:**
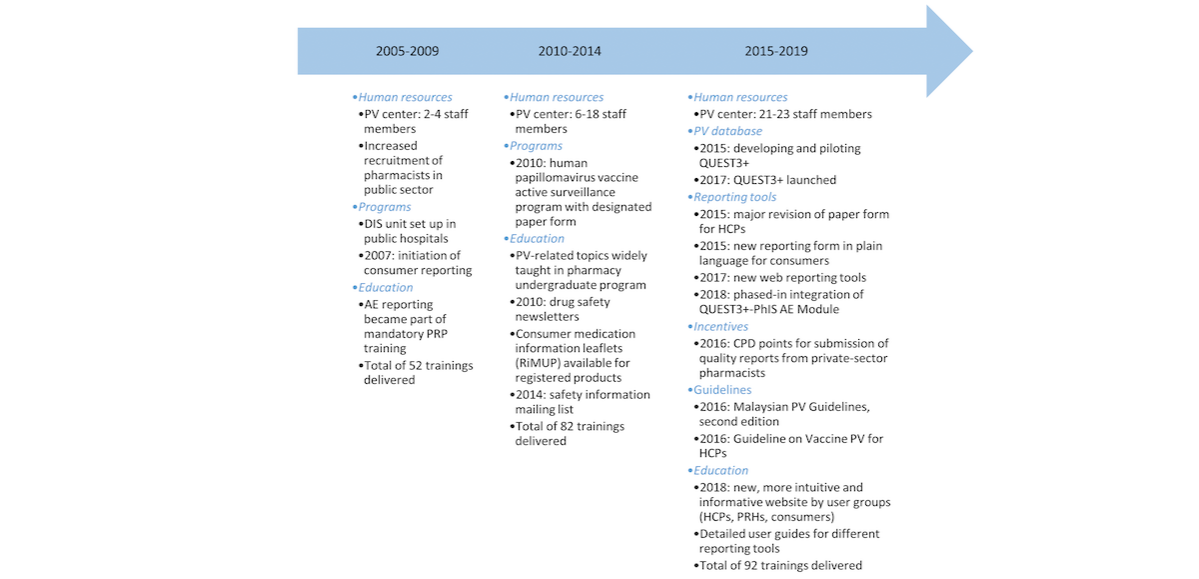
Key strategies and interventions implemented to improve adverse event (AE) reporting in Malaysia between 2005 and 2019. CPD: continuing professional development; DIS: drug information service; HCP: health care professional; PhIS: pharmacy hospital information system; PRH: product registration holder; PRP: provisionally registered pharmacist; PV: pharmacovigilance; RiMUP: *risalah maklumat ubat untuk pengguna*.

In Malaysia, various strategies and interventions were implemented over the 15 years with the intent of improving AE reporting, as summarized by 5-year period in Figure 4 [7,61-65]. While the impacts of most interventions are usually multifaceted at the national level and challenging to measure with limited quantitative information, there is a particular interest in understanding the influence of staffing levels at the PV center, the introduction of a new PV database, and enhancements to reporting tools on reporting quality at different time points.

**Figure 5:**
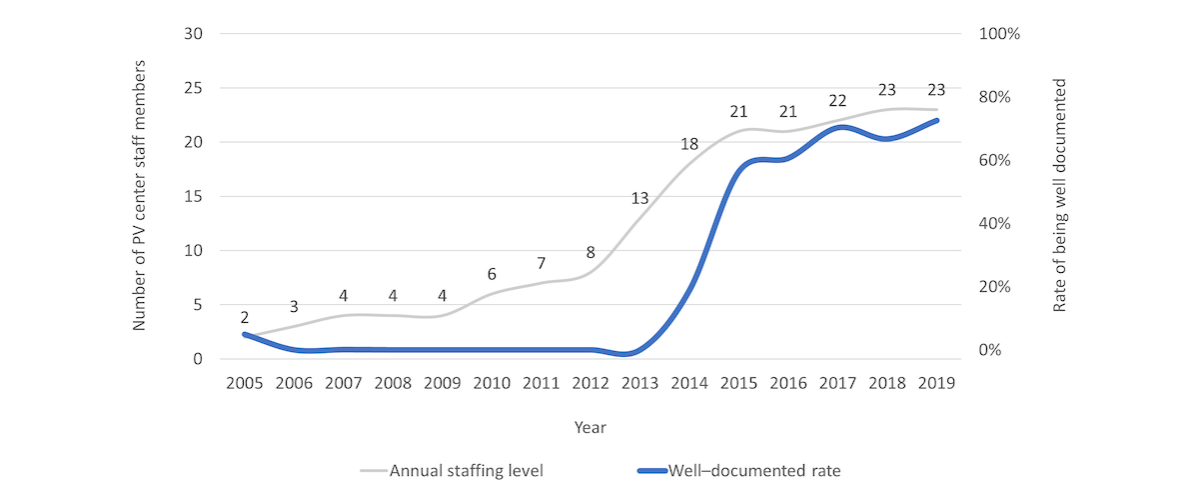
Annual trends in pharmacovigilance (PV) center staffing levels and rate of reports being well documented (2005-2019).

**Figure 6:**
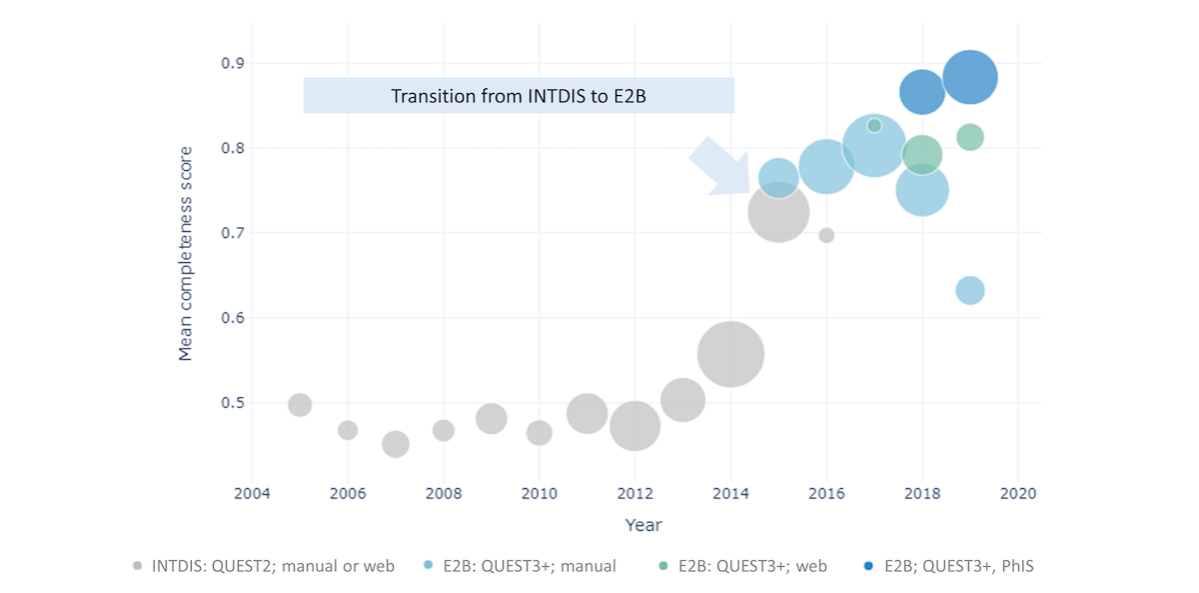
Mean completeness score and report count by submission format, pharmacovigilance database, and means of reporting yearly from 2013 to 2019. The size of the bubble corresponds to the report count. INTDIS: International Drug Information System; PhIS: pharmacy hospital information system.

**Figure 7:**
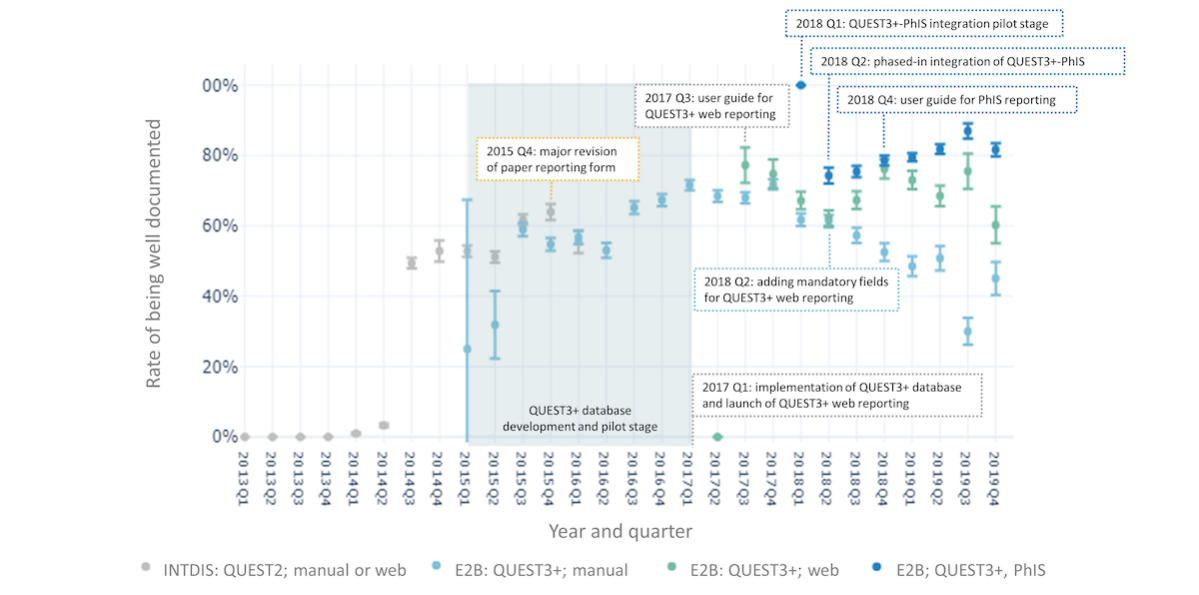
Rate of reports being well documented by submission format, pharmacovigilance database, and means of reporting quarterly from 2013 to 2019. 95% CI error bars (equivalent to 1.96×SE) were constructed. INTDIS: International Drug Information System; PhIS: pharmacy hospital information system; Q: quarter.

Figure 5 shows the annual trends in PV center staffing in relation to rates of reports being well documented. Figure 6 depicts how the transition in report submission format (from INTDIS to E2B) and reporting means correlated with report quantity and average completeness. In Figure 7, we focus on the rates of reports being well documented before and after the implementation of the new PV database (QUEST3+) and key enhancements to reporting tools since 2015. Information about reporting means was not available for INTDIS reports collected from the historical QUEST2 database. We further examined the influence and popularity of different reporting means among various reporters following the official launch of QUEST3+ and new web reporting tools in the first quarter of 2017 (Figure S4 in Multimedia Appendix 2).

#### Average Completeness of Individual Dimensions for Malaysian AE Reports

**Figure 8:**
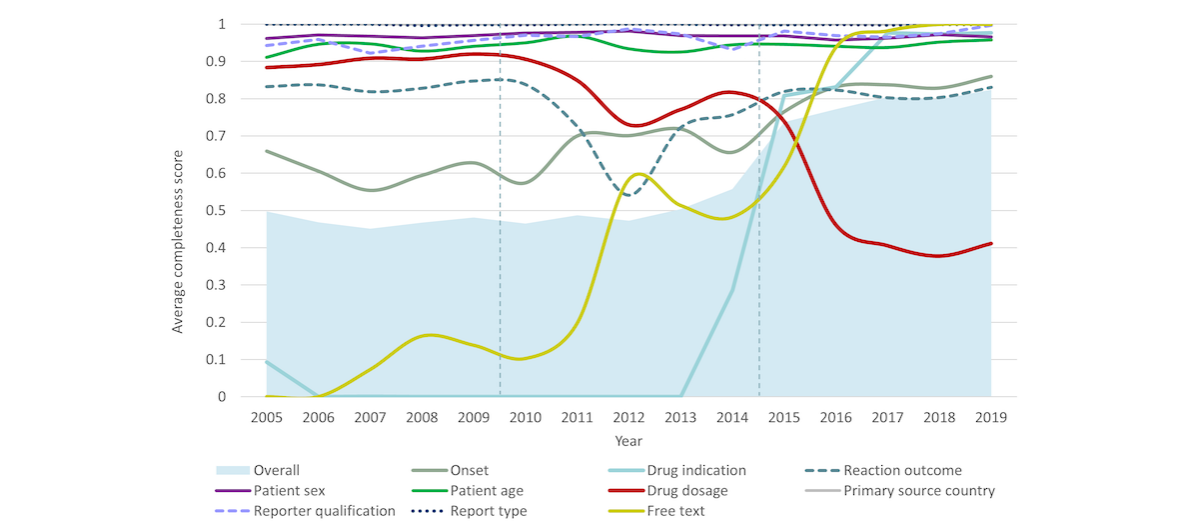
Trends in the distribution of average completeness scores for Malaysian reports in VigiBase over the study period. The shaded area indicates overall completeness; the line represents the individual dimension.

Figure 8 illustrates the trends in the average completeness of the individual dimensions for Malaysian reports in VigiBase. The dimensions for report type, primary source country, reporter qualification, and patient age and sex were consistently the most completed. Completeness of free text and drug indication improved from zero in the earlier period of 2005 to 2009 to >0.9 in recent years. An uptrend in improvements was also observed for reaction onset. Completeness for reaction outcome dipped in 2011 to 2012 but subsequently rebounded to >0.8. Unexpectedly, we observed a noteworthy drop in drug dosage completeness since 2010. The average completeness of each individual dimension for vigiGrade was evaluated for E2B subsets (Figure S5 in Multimedia Appendix 2).

## Discussion

### Overview of Principal Findings

In our study, we used a comprehensive approach to examine the factors influencing the quality of Malaysian AE reports and decipher the temporal trends and interventions that shaped the Malaysian PV landscape from 2005 to 2019. In the first part of our study, by harnessing a data-driven approach that encompassed ML-based feature selection and analysis, we identified the key features that predict the status of Malaysian reports in VigiBase being well documented. Our hybrid feature selection helped in mitigating the risks of overfitting and unstable interpretability that are commonly associated with high variance [8,9,40] and resulted in a robust and valid RF model, as evidenced by the excellent classification performance (>90%) across all training, validation, and test sets for the E2B subset that reflects recent patterns of Malaysian reports (Table 1). While the model for the highly imbalanced INTDIS subset (containing only 10,691/63,943, 16.72% of well-documented reports) demonstrated satisfactory performance, with recall, precision, and *F*_1_-scores of >70%, the model faced overfitting. This issue, evident from the decreased validation and test performance, has been acknowledged as a limitation in our study. The black-box RF model was made interpretable using SHAP values that, in agreement with human intuition, did not contradict the findings from the time-series analyses. To the best of our knowledge, this is the first study to use state-of-the-art interpretable ML methods to obtain insights concerning the key factors contributing to AE report quality in the PV field. Supplemented with insights drawn from our time-series and descriptive analyses, we summarized the identified features associated with well-documented Malaysian reports under 3 main themes: administrative; sender and reporter; and reaction, drug, and patient.

In the second part of our study, our extensive time-series and descriptive analyses illuminated the notable progress Malaysia has made in both the quantity and quality of AE reports over the years. We delved further into the chronological trends and characteristics of Malaysian AE reports, outlined the key strategies and interventions implemented at 5-year intervals, and tracked the influence of interventions of interest. These findings offer valuable insights into the multifaceted strategies and interventions that have driven enhancements in Malaysian AE reporting quality. Finally, by focusing on individual dimensions for vigiGrade completeness scoring, we not only gained additional insights into the specific characteristics and aspects of report completeness within the Malaysian context but also pinpointed systematic data quality issues that warrant further attention and improvement work.

### Factors and Characteristics Associated With Well-Documented Malaysian Reports

#### Administrative

Our ML analysis distinguished PV center staffing level as the most important factor positively associated with well-documented INTDIS reports over 12 years between 2005 and 2016 (Figure S3 in Multimedia Appendix 2 and Figure 2A). Previous studies [66-68] have also highlighted manpower at national PV centers as a barrier to functional and sustainable PV systems. During the initial stage (2005-2009), only 2 to 4 staff members handled all PV operations in Malaysia (Figure 5). As the number of reports grew rapidly, the center struggled with a backlog of reports. The center swiftly grew from 8 to 21 staff members within 3 years (2012-2015) alongside the expansion of PV functions and task specializations, which coincided with a notable improvement in the completeness of reports received (Figures 3 and 5). Compared to the period from 2005 to 2009, more training workshops were delivered from 2010 to 2019 (Figure 4) to enhance staff and reporter competencies. As the staffing level and rate of reports being well documented became relatively stable afterward (Figure 5), the influence of PV staffing levels appeared less distinctive for E2B reports (Figure 2B). This could be due to some staff members focusing on other PV duties such as signal detection and assessment, risk management, and risk communication rather than AE processing. It is worth noting that the transition to E2B may have also obfuscated the influence of increased staffing on report completeness. While less distinctive in our model, we cannot exclude that other centers with E2B reports and low staffing may still face overall low report completeness.

The capabilities and scalability of PV databases are essential for effective data collection and management [5]. Integrating electronic reporting into hospital information systems has been reported to reduce duplicate work and effectively improve AE reporting [69]. In Malaysia, the new QUEST3+ database was later integrated with the AE reporting module of a centralized PhIS implemented at Malaysian public hospitals and clinics, enabling the automatic input of reporter and sender information and improved reporter accessibility to patient, drug use, and regulatory information (eg, product registration number and batch number). Our ML analysis of E2B subsets (Figure 2B) revealed that PhIS-integrated reporting positively contributed to well-documented Malaysian reports, whereas web reporting had an unexpected negative association. The volume of PhIS reports surpassed other means in 2019 (Figure 7 and Figure S4 in Multimedia Appendix 2) and maintained the highest reporting quality. Web reporting was initially introduced in 2000, but due to unstable systems and slowly developing IT infrastructures at some public health facilities in the last decade, most reports before 2015 were submitted manually via postage, fax, or email. After mandatory fields were added, web reporting recorded a rate of >70% of reports being well documented, but it later declined to 60%. A similar but larger declining trend was also observed with manual reporting (Figure 5). These declines corresponded to the shift to PhIS reporting by public-sector pharmacists and were likely associated with other HCPs and consumers (Figure S4 in Multimedia Appendix 2). Our findings highlight that electronic reporting tools serve as ad hoc support for well-trained reporters but IT infrastructure maturity and widespread user acceptance are required for success. This is in line with a recent realist review [70] asserting that technological interventions alone, without capacity building, have little or no impact on health data quality.

#### Sender and Reporter

Our study revealed that the greatest proportion of well-documented Malaysian reports, amounting to 91.54% (52,066/56,877), originated from public health facilities overseen by the MOH (Table 3). Among the well-documented reports, 98.15% (55,825/56,877) were submitted by HCPs. This finding aligns with those of previous studies [71-73] conducted across different regions of Malaysia, which consistently highlight that most HCPs recognize AE reporting as part of their professional obligations. In Malaysia, consumer-generated reports constituted only 0.14% (78/56,877) of the well-documented reports, which stands in contrast to countries such as Denmark and Norway where three-fifths of well-documented reports came from consumers or non-HCPs [6].

Our observations revealed that Malaysian pharmacists generated 70.85% (40,295/56,877) of well-documented reports (Table 3), with most serving the public health sector [63]. Specifically, we identified public specialist hospitals and pharmacists as key features that positively contributed to the well-documented Malaysian E2B reports (Figure 2B). It is noteworthy that, during the earlier stage (2005-2009), pharmacists’ reports exhibited the poorest quality and were recognized as a key factor predicting not well-documented INTDIS reports from 2005 to 2016 (Figure 2A). Nonetheless, over the years, following increased recruitment in public health services [74,75] and multifaceted initiatives aimed at strengthening pharmacists’ roles and skills (Figure 4), pharmacists have become an integral part of AE monitoring in Malaysia. Almost 9 in 10 public hospital pharmacists in Malaysia have reported at least one AE in the past [76].

Malaysia presents a unique scenario in which pharmacists play a leading role in PV activities, distinguishing it from global trends, in which physicians contribute nearly two-thirds of well-documented reports [6]. The Malaysian context aligns with findings from a Spanish study in which pharmacists reported a great majority of the AEs due to the integration of PV into routine hospital pharmacy practices [77]. Similar observations were made in a pharmacist-led AE monitoring and management model in China, where pharmacists provided higher-quality reports among all HCPs [14]. Within Malaysian public hospitals, the pharmacist-led Drug Information Service (DIS) unit is responsible for facility-level PV activities, including responding to queries related to AEs; disseminating safety information; and compiling, verifying, and submitting AE reports to the NPRA [65,78]. In addition to direct detection and reporting by pharmacists, a collaborative mechanism exists within public health facilities where physicians and other HCPs are aware of the role of pharmacists in monitoring and reporting the AEs detected during clinical rounds or discussions. Moreover, we observed that over half (19,188/33,559, 57.18%) of the well-documented E2B reports made by pharmacists came from public specialist hospitals. These reports were believed to have benefited from the input of specialist physicians, suggesting a positive contribution of collaborative efforts among HCPs in enhancing the quality of AE reports.

In contrast, reports from PRHs and other HCPs (including regulatory affairs officers, clinical trial associates, nurses, and medical assistants) were flagged as the key features negatively associated with Malaysian report completeness (Figure 2B). These findings are consistent with the features observed in the United States [15], Brazil [16], Spain [17], South Korea [18], and Japan [19,20]. Of note, the NPRA classified the reports from PRHs as reported by other HCPs when the primary reporter was unknown. Among 7833 E2B reports from PRHs, only 778 (9.93%) reports were well documented (Table S1 in Multimedia Appendix 2), with an overall completeness score of 0.39 (Figure S5 in Multimedia Appendix 2). Information regarding drug dosage, reaction onset, reaction outcome, and patient age was most incomplete in Malaysian reports from PRHs. This could be attributed to the lack of a robust PV culture among PRHs. Existing literature [6,79] suggests that PRHs might prioritize submitting a report to fulfill pharmaceutical legislation [80] that mandates that PRHs report any suspected AEs within strict timelines even when minimal information is available. There could also be instances in which the primary reporter did not provide consent for follow-up. Conversely, it is conceivable that pharmacists and physicians serving at health facilities were more motivated to make a clinically meaningful report even on a voluntary basis [17,79,81].

#### Reaction, Drug, and Patient

As AE reporting is highly dependent on individual motivation [5], we were interested in understanding whether the nature of drugs and reactions affects the quality of reports. While a report may involve more than one drug or reaction, most studies on AE report quality have not assessed all drugs and reactions reported in a case. Toki and Ono [21] examined only the primary suspected drug in a multivariable logistic regression model, whereas Araujo et al [22] and Masuka and Khoza [23] evaluated a specific drug group using simple univariable analysis. Other studies have evaluated only case-level information, such as case seriousness [14,18,24-26], fatal outcome [15], and causality [18]. As we converted drug-reaction pairs to case-level data, we evaluated the influence of drug- and reaction-related factors on overall report completeness for all reported suspected and interacting drugs.

Our ML analysis of the E2B subset (Figure 2B) revealed that a case where the reaction abated following a drug dechallenge (ie, positive dechallenge) was the primary key feature associated with a well-documented report. While information on drug dechallenge was unknown in most cases (Table S1 in Multimedia Appendix 2), it is possible that a positive dechallenge may have strengthened the reporter’s confidence in the drug-reaction causal relationship and, thus, the motivation to construct a clinically meaningful report. While our findings suggest that positive dechallenge may have motivated more complete reports, there is no supporting study on this. It is important to note that reports that are well documented by vigiGrade standards might also tend to have a positive dechallenge. In other words, it may be that vigiGrade tends to flag those reports that have a positive dechallenge as well documented.

As expected, cases containing information on time to onset were more likely to be well documented as the onset dimension incurs the highest penalty of 50% for missing data and 30% if the uncertainty exceeds 1 month [6]. Our findings suggest that cases with a shorter (ie, <1 day) time to onset, dosing interval, and duration of drug use were most likely to be well documented. This observation might be attributable to better recall and description of events occurring within a shorter time frame following drug use or to greater reporter confidence in the drug-reaction relationship due to stronger temporal association and a lower likelihood of confounding factors. On the other hand, reports that involved reactions lasting 1 to 6 days tended to carry more information compared to those that involved reactions lasting <1 day or >6 days. A competing hypothesis is that reactions occurring within this time frame allow for sufficient time for more observation or data gathering while still being easily observed and described by patients and HCPs. Nevertheless, further research is needed to determine the specific factors contributing to the observed differences in report quality for cases with varying time to onset, dosing intervals, and durations of drug use.

Antibiotics for systemic use (ATC code J01) emerged as a key feature favorably contributing to well-documented E2B reports. First, it could be attributed to the baseline reporting patterns, where systemic antibiotics were the most commonly reported drug group in Malaysia, with over one-fifth of AE reports involving at least one systemic antibiotic (Table S1 in Multimedia Appendix 2). Second, this observation might suggest that Malaysian HCPs exercise heightened caution when using anti-infectives, leading to a higher likelihood of detecting AEs related to anti-infectives with higher report completeness. According to a study from a Malaysian infectious disease hospital [65], most inquiries (37.8%) received by the DIS unit concerned anti-infective drugs (ATC code J), which included other β-lactam antibacterials (ATC code J01D), direct-acting antivirals (ATC code J05A), and penicillins (ATC code J01C), with the largest proportion of the inquiries pertaining to their AEs and pediatric dosage adjustments. Although this observation could be expected in an infectious disease hospital, it also highlights the role of pharmacist-led DIS in AE monitoring and reinforces our previous discussion regarding pharmacists working in public health facilities tending to submit more complete reports. Trainings by the NPRA often prioritized pharmacists working in DIS units, who then conducted echo training for HCPs in their respective health facilities.

In addition, our analysis revealed a positive association between reports marked as serious and well-documented Malaysian reports (Figure 2B), consistent with previous studies from France [25,26], China [14,24], and South Korea [18] that highlighted higher completeness for serious reports. Previous research has also indicated that Malaysian HCPs prioritize reporting serious AEs [71]. The heightened gravity and potential consequences of these cases might prompt reporters to exercise greater diligence in ensuring reporting quality, including PRHs who are subjected to stricter reporting timelines for serious cases. Fatal outcomes were not flagged as the key feature contributing to Malaysian reports being well documented, likely due to their low prevalence (1048/68,795, 1.52%; Table S1 in Multimedia Appendix 2). Nonetheless, Malaysian fatal reports had a low overall completeness of 0.55 compared to 0.80 for nonfatal reports (Figure S5 in Multimedia Appendix 2). Another observational study evaluating reports submitted to the US Food and Drug Administration [15] also found that cases of patient deaths had the lowest completeness scores across reporting sources. This could be attributed to the absence of medical terminology describing the cause of death or indicate an investigation into a potential drug involvement.

Reactions under the HLGTs *product quality, supply, distribution, manufacturing,* and *quality system issues*, of which 97.78% (2242/2293) were related to product substitution issues and 91.41% (2096/2293) were sourced from public health facilities primarily by pharmacists, were captured as the key feature that negatively contributed to E2B reports being well documented. Subsequent investigations revealed that product substitution issues were most prevalent in 2018 (1355/2242, 60.44%) and primarily involved brand switching between 2 generic products: amlodipine (1012/2242, 45.14%) and perindopril (291/2242, 12.98%). Among these, only 24.8% (251/1012) and 32.6% (95/291) of the reports involving amlodipine and perindopril, respectively, were well documented. In the Malaysian public health care sector, drugs are procured through 3 distinct mechanisms: a national concessionaire, national tenders, and direct purchases by health facilities for items not covered by the former 2 mechanisms [82]. However, in situations in which a product substitution issue is suspected and public health facilities need to directly procure alternative products for items already listed in the former 2 mechanisms, AEs or product complaints must be submitted as justification. It is believed that the reporters might submit reports containing only minimal information solely to comply with the drug procurement procedure.

Another key negative feature identified in the E2B subset was the presence of adolescent patients (aged 12-17 years). In comparison, reports involving adult patients (aged 18-44 years) and midlife adult patients (aged 45-64 years), which comprised the largest proportion of Malaysian reports, tended to be well documented. In South Korea, overall reports involving children and adolescents (aged 0-19 years) were negatively associated with being well documented in comparison to the older adult group (aged ≥65 years), whereas reports involving adults (aged 19-65 years) had a positive association [18].

### Trends in Malaysian AE Reporting Quality Between 2005 and 2019

Building on the preceding discussion on the factors and characteristics associated with well-documented Malaysian AE reports, we expanded our scope to the chronological progression of AE reporting in Malaysia from 2005 to 2019. Our analyses underline that policy changes, continuity of education, and human resource development laid the foundation for a functional and sustainable SRS in Malaysia. Meanwhile, advancements in technological infrastructure, PV databases, and reporting tools contributed to the observed increase in both the quantity and quality of AE reports. These findings echo the expert-recommended 4-tier hierarchy of needs to achieve systemic capacity building for PV [83]—progressing from structures, systems, and roles to staff and infrastructure to skills to tools.

Malaysia, with its SRS governed within an established legal and regulatory framework, has historically struggled with challenges of underreporting and poorly reported AEs, as evidenced in Figure 3. In an effort to establish a functional and sustainable PV system, Malaysia placed early priorities on cultivating a reporting culture among HCPs and strengthening national PV capacities through collaborative efforts involving multiple stakeholders (Figure 4). Among them were policy changes to strengthen pharmacists’ role in AE monitoring, increased recruitment of public-sector pharmacists and PV staff, active surveillance programs for targeted medicinal products, public awareness campaigns, and continuity of PV education to HCPs from undergraduate and preservice to at-service levels [7,61-63,65,74,75]. These initiatives were consistent with the existing literature, which emphasizes that multifaceted strategies and interventions work more synergistically to improve AE reporting than a single intervention [79,84,85]. Notably, our findings from the ML analysis suggest a positive association between higher staffing levels at the PV center and well-documented INTDIS reports, which could underscore the potential need for capacity building in the early phase of PV implementation.

As PV activities in Malaysia attained a higher level of maturation, the NPRA began to put greater emphasis on improving report quality. Comparative studies examining reporting forms from various countries have consistently highlighted that the Malaysian paper reporting form captures the most comprehensive information [86,87]. In response to the influx of reports observed in 2014 (Figure 3), the NPRA set their efforts on enhancing AE reporting tools and processing capabilities (Figure 4). Enhancements were made to the paper reporting form in 2015, including the addition of structured checkboxes and a reporting guide to ensure that more complete and harmonized clinical information could be obtained for subsequent causality assessment [27,88]. Concurrently, the NPRA began developing and piloting QUEST3+, an upgraded regulatory database system that marked a new submission format to the UMC—transitioning from INTDIS to E2B (Figure 6). The official launch of the QUEST3+ database took place in January 2017, replacing the historical QUEST2 database. Alongside these paradigm shifts, the NPRA also revamped and relaunched its web reporting tool for HCPs and introduced a new plain-language web reporting tool specifically for consumers (ConSERF). With the maturation of IT infrastructures, in 2018, the QUEST3+ database was integrated in phases with the centralized PhIS across Malaysian public health facilities. Reporting guides, drop-down lists, and validation alerts were also added to web and PhIS-integrated reporting tools to enhance the completeness and consistency of the collected data. Interestingly, as previously discussed, our comparative findings regarding PhIS-integrated tools used by well-trained pharmacists and new web reporting tools likely used by other HCPs and consumers highlight the complementary role of electronic reporting tools as ad hoc aids for well-trained reporters, whereas the effectiveness of these tools in improving AE reporting also relies on the maturity of IT infrastructures and their acceptance by users.

As a consequence of continuous efforts to strengthen PV capacities and technological advancements, Malaysia has seen considerable improvements not only in the quantity but also in the quality of reports. From 2015 to 2019, approximately two-thirds of Malaysian reports were well documented compared to approximately 1 in 5 reports from the rest of the world [28]. It is worth noting that, while overall completeness improved after the transition to the E2B submission format in 2015, our investigations revealed that low completeness in drug dosage (Figure 8) was systematically confounded by miscoding errors during report conversion to E2B-XML files before report transmission to VigiBase and, thus, was comparatively lowest in all subsets (Figure S5 in Multimedia Appendix 2). As a consequence of missing “number of unit in the interval” and miscoded “number of separate dosages,” the drug dosage dimension for a Malaysian report in E2B format was penalized when the total daily dose for a case could not be calculated from the specified fields [29,89]. Similar to global reports [6], Malaysian E2B reports carried more administrative information, such as report type followed by reporter qualification and patient characteristics (ie, sex and age), but less drug- and reaction-related information, such as drug dosage (despite the aforementioned confounding), reaction onset, and reaction outcome. In contrast to global reports [6], the inclusion of mandatory fields in electronic reporting tools led to a higher completeness of drug indication and free-text narratives in Malaysian reports. Reports from the literature and other sources, made by other HCPs, and submitted by PRHs had the lowest overall completeness scores (<0.5). Fatal reports and those from community pharmacies or other public services also tended to contain less information (<0.6).

### Limitations

Our study is constrained by the limitations inherent to cross-sectional observational data and ML analysis, where causal reasoning and statistical inference cannot be determined [54]. The features identified in our study should be understood as predictors associated with well-documented reports but not as causal factors. Owing to the assumptions that multifaceted interventions often work synergistically and that control groups are frequently absent in nationwide implementation [79], the exact impact of individual interventions on reporting quality cannot be determined. As such, our study serves as an exploratory analysis, and the highlighted features offer a starting point for further in-depth review.

Our study faced challenges with the class imbalance inherent to the INTDIS data set, which heightened the risk of model overfitting. While undersampling improved the balanced performance of precision and recall, it could introduce new biases. Given that the INTDIS format is now obsolete in Malaysia, our focus is shifting toward the more recent E2B features.

Our models did not include causality information for several reasons. AE reports received by the NPRA and VigiBase come from a variety of sources, and the probability that the suspected AE is drug related is not the same in all cases [90]. Reporters and senders might use different methods for assessing causality, such as Naranjo probability scores and WHO-UMC causality categories, which were not available. In addition, it is important to note that causality may change as knowledge expands, and the UMC does not validate the causality assessments of the received reports.

Our data set is also constrained by the timeliness of report submission from QUEST to VigiBase and did not include all the reports received by the NPRA by December 31, 2019. As the systematic data quality issues uncovered in our study have already been communicated to the NPRA, follow-up work is underway to address these issues. Therefore, the findings of this study will not be representative of the future completeness scores of Malaysian reports in VigiBase. This also implies that the key features identified in our study were subject to multiple systematic biases, which are typically encountered when using real-world data [90,91].

### Conclusions and Future Work

By using a data-driven approach and the vigiGrade method, we pinpointed the trends and milestones of the Malaysian AE reporting system and demonstrated how the country has striven to contribute large numbers of high-quality reports to global PV. Our work also highlights the vigiGrade method by the UMC as an effective tool for monitoring the quality of AE reports and aiding countries in evaluating to enhance reporting. Our multidimensional perspective on AE reporting trends and strategies in Malaysia, informed by data-driven insights, underlines the complexity and evolving nature of the SRS and the importance of continual improvement for global PV.

Using interpretable ML methods, we identified specific features that were positively associated with Malaysian AE reports being well documented. Notable factors include higher PV center staffing for INTDIS reports, reaction abated upon drug dechallenge, reaction onset or drug use duration of <1 week, dosing interval of <1 day, reports from public specialist hospitals, reports by pharmacists, and reaction duration between 1 and 6 days for recent E2B reports. Conversely, reports from PRHs and other HCPs indicated areas for potential improvement in the quality of Malaysian reports. These identified features could potentially serve as a basis for future research and strategies aimed at improving PV practices, thus improving drug safety surveillance and, ultimately, public health outcomes.

Furthermore, our time-series analysis showcased how Malaysia has built up and strengthened its PV capacity via multifaceted strategies and interventions to enhance both the quantity and quality of AE reports. Policy changes, continuity of education, and human resource development have all contributed to the foundation for a functional and sustainable SRS in Malaysia, whereas advancements in technological infrastructure, PV databases, and reporting tools concurred with the rise in both the quantity and quality of AE reports. These findings resonate with the expert-recommended 4-tier hierarchy of needs for systemic PV capacity building—from structures, systems, and roles to staff and infrastructure to skills to tools [83,92].

Building on our findings on Malaysia’s progress in AE reporting and factors identified for report quality, we propose several areas for future work. To understand how and in what measure the findings from the time-series analysis contributed to the completeness of Malaysian reports, viewing the interventions set up by the NPRA as complex [93]—targeting multiple individuals or a wide range of behaviors and involving multiple interacting components—could be instrumental. Future evaluations may use this newly updated framework for complex intervention research [94].

Our findings revealed that the private health sector, including PRHs, private hospitals, private clinics, and community pharmacies, exhibited suboptimal contributions. This highlights persistent challenges pertaining to underreporting and unsatisfactory report quality in these sectors, necessitating further research into understanding behavioral or organizational barriers for developing targeted interventions [95,96]. Considering that preservice and in-service trainings often do not adequately prepare HCPs for data-related tasks [96], stronger stakeholder coordination and collaboration are imperative for continuous competency-based training and fostering an effective data use culture across health systems [95]. Regular feedback on reporting performance could be considered to facilitate self-monitoring among all senders.

Sustainable improvement in surveillance data quality and use requires a whole-systems approach encompassing governance, people, tools, and processes [95]. Given that data quality is highly reliant on their collection at health facilities, future work can prioritize people and environments essential for functional information systems as well as validation upon data entry to ensure completeness, accuracy, and consistency [88]. Our identification of systematic data quality issues highlighted a gap in data-driven continuous quality improvement [95], underscoring the need for internal quality assurance procedures for AE data management and transmission, including routine systematic checks and periodic in-depth reviews [88,97].

Looking ahead, as Malaysian reports currently use the E2B(R2) format, future efforts can navigate toward transitioning to the E2B(R3)-compliant database and reporting tools as the inclusion of null flavors in the E2B(R3) format helps address missing information by explaining data absence. Future work on data governance could explore leveraging automation, ML, and natural language processing to improve the overall efficiency and quality of AE data collection, processing, and management [98,99].

## Appendix

Multimedia Appendix 1 List of definitions or operational definitions.

Multimedia Appendix 2 Supplementary figures and tables.

Multimedia Appendix 3 Literature review on quality of reports in the spontaneous reporting system.

Multimedia Appendix 4 Feature selection.

Multimedia Appendix 5 Tree-based machine learning models and interpretable machine learning.

## Abbreviations

AE: adverse event
ATC: Anatomical Therapeutic Chemical
DCA: Drug Control Authority
DIS: Drug Information Service
HCP: health care professional
HLGTs: High-Level Group Terms
ICH: International Council for Harmonisation of Technical Requirements for Pharmaceuticals for Human Use
ICSR: individual case safety report
INTDIS: International Drug Information System
MADRAC: Malaysian Adverse Drug Reactions Advisory Committee
MedDRA: Medical Dictionary for Regulatory Activities
ML: machine learning
MOH: Ministry of Health
NPRA: National Pharmaceutical Regulatory Agency
PhIS: pharmacy hospital information system
PRH: product registration holder
PV: pharmacovigilance
RF: random forest
SHAP: Shapley additive explanations
SRS: spontaneous reporting system
UMC: Uppsala Monitoring Centre
WHO PIDM: World Health Organization Programme for International Drug Monitoring
WHO: World Health Organization

## Data Availability

The data sets generated during and analyzed during this study are not publicly available due to the data protection policies of the Uppsala Monitoring Centre and the National Pharmaceutical Regulatory Agency. Data requests should be made directly to the institutions.
