# Supplementary material for: Data-Driven Identification of Factors That Influence the Quality of Adverse Event Reports: 15-Year Interpretable Machine Learning and Time-Series Analyses of VigiBase and QUEST
Source: JMIR Med Inform. 2024 Apr 3;12:e49643. doi: 10.2196/49643 (PMC11024759; doi:10.2196/49643)
Supplement: Multimedia Appendix 1 [file medinform_v12i1e49643_app1.pdf]

Multimedia Appendix 1

List of definitions or operational definitions

Definitions

Adverse drug reaction (ADR) is a harmful effect suspected to be caused by a drug. This term has been used quite loosely to include all kinds of adverse events, many of which are not ‘reactions’ in the strict sense at all, and have not been subject to any assessment of causality. The term is properly reserved for late-stage analysis when the association between a medicine and an adverse effect has moved beyond 'unmeasurable' or 'uncertain' [1].

Adverse event (AE) is any negative or harmful occurrence that takes place during treatment, that may or may not be associated with a medicine. Note. A fall could be such an event that may – or may not – have any association with a medicine [1].

Individual case safety report (ICSR) are reports sent by health professionals or patients when an adverse effect has occurred in a patient taking one or more medicines. These have also been referred to as adverse drug reaction (ADR) reports or adverse event (AE) reports [1].

Machine learning (ML) is the use and development of computer systems that are able to learn and adapt without following explicit instructions, by using algorithms and statistical models to analyze and draw inferences from patterns in data [2]. Similarities in concepts and nomenclatures across ML and traditional medical statistics were outlined as below [3, 4]:

Machine learning	Comparable statistical concept
Feature	Independent variable
Label/Class/Outcome	Dependent variable
Training/Learning	Model estimation
Recall	Sensitivity
Precision	Positive predictive value
Area under receiver operating curve (AUROC)	C-statistic

QUEST system is a consolidated repository of regulatory controls over the pharmaceutical and cosmetic products in Malaysia, including registration, variations, licensing, compliance, quality control, clinical trials, and surveillance. QUEST is governed by the National Pharmaceutical Regulatory Agency (NPRA) which is under the purview of Ministry of Health of Malaysia. QUEST 3+ is the latest system version [5].

Spontaneous reporting system (SRS), also referred to as pharmacovigilance reporting system postmarketing safety surveillance, is the core data-generating system of pharmacovigilance [1]. Pivotal SRS databases are almost exclusively governed by government or multilateral health organizations under a legal and regulatory framework. The heterogeneity of the primary sources of data (e.g., healthcare professionals (HCPs), patients, clinical trials, literature, legal representatives, etc.) and disparate safety concerns (e.g., AEs from medication errors or quality defects) from SRS produce a multi-dimensional and huge real-world dataset [1, 6].

Spontaneous reports, in this study, refer to all kinds of reports submitted to the spontaneous reporting system.

VigiBase is the unique World Health Organization (WHO)'s global database of Individual Case Safety Report (ICSRs). It is the single largest drug safety data repository in the world that contains reports of suspected AEs of medicines submitted by member countries of the WHO Programme for International Drug Monitoring [1].

Operational Definitions

vigiGrade Completeness Score, C: The vigiGrade completeness scoring method, which was developed by UMC, is a multidimensional measure of the amount of information on reports. It quantifies how complete the information on the report is, based on a selection of ICH-E2B fields submitted from national databases to VigiBase. Each report field is assigned a weight based on its importance in regard to causality assessment. The vigiGrade completeness score ranges from 0.07 to 1. It starts at 1 (which represents a fully complete report), and for every missing dimension, the corresponding penalty factor in **Table 1** is applied, as illustrated in **Figure 1** [7]. This measure of completeness is, however, independent of whether the information establishes a causal relationship between drugs and events.

Table 1: Overview of the dimensions accounted for in the vigiGrade completeness score [7]

Dimension	Description	Considerations	Penalty (%)
Time-to-onset	Time from treatment start to the suspected ADR	Imprecise information penalised if there is ambiguity as to whether the drug preceded the adverse event; by 30 % if the uncertainty exceeds 1 month, 10 % otherwise	50
Indication	Indication for treatment with the drug	Penalty imposed if information is missing or cannot be mapped to standard terminologies such as ICD or MedDRA	30
Outcome	Outcome of the adverse event in this patient		30
Sex	Patient sex	'Unknown' treated as missing	30
Age	Patient's age at onset of the suspected ADR	Age 'unknown' treated as missing<br>10 % penalty imposed if only age group is specified	30
Dose	Dose of the drug(s)		10
Country	Country of origin	Supportive in causality assessment since medical practice and adverse reaction reporting vary between countries	10
Primary reporter	Occupation of the person who reported the case (e.g. physician, pharmacist)	Supportive in causality assessment since the interpretation of reported information may differ depending on the reporter's qualifications<br>'Unknown' penalised as missing information, but 'other' not penalised	10
Report type	Type of report (e.g. spontaneous report, report from study, other)		10
Comments	Free-text information	Uninformative text snippets excluded	10

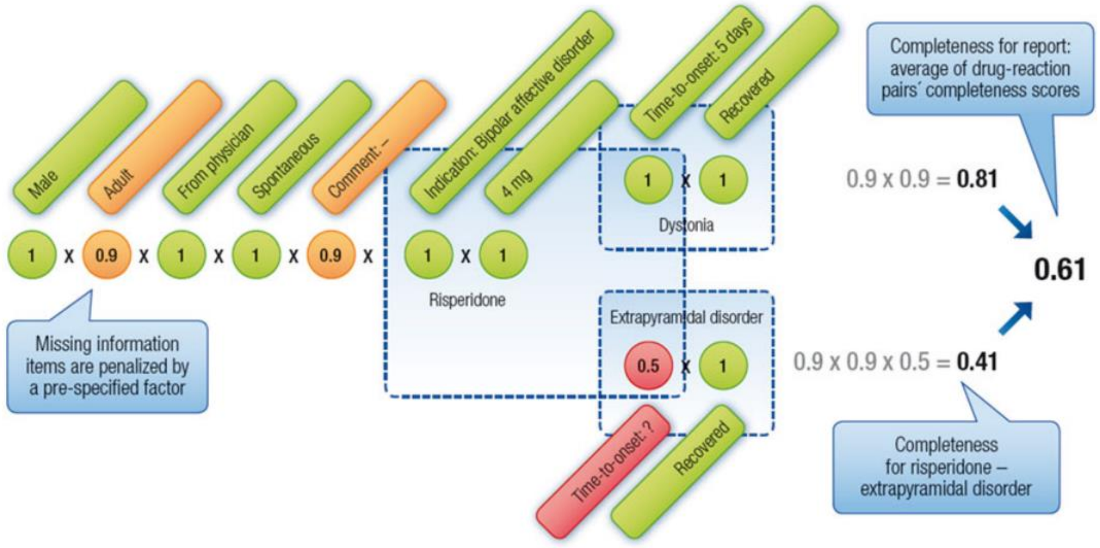

Figure 1: An example of how the vigiGrade completeness score is calculated for a report [7]

Well-Documented Report: A report is classified as well-documented if the vigiGrade completeness score is > 0.8. This threshold requires all the important and essential dimensions, i.e., 30 % penalty, to be provided and allows at most two of the supportive dimensions, with 10 % penalty, to be missing [7].

vigiMatch: The vigiMatch method is an algorithm that was developed by UMC for automatic duplicate detection in VigiBase. It detects suspiciously similar pairs of individual case safety reports, using probabilistic pattern matching [1].

References:

1. Uppsala Monitoring Centre. URL: https://www.who-umc.org/ [accessed 2021-05-22]
2. Merriam-Webster. URL: https://www.merriam-webster.com/dictionary/machine%20learning [accessed 2024-03-18]
3. Wiemken TL, Kelley RR. Machine learning in epidemiology and health outcomes research. Annu Rev Public Health. Apr 02, 2020;41:21-36. [FREE Full text] [doi: 10.1146/annurev-publhealth-040119-094437] [Medline: 31577910]
4. Stevens LM, Linstead E, Hall JL, Kao DP. Association between coffee intake and incident heart failure risk. Circ Heart Failure. Feb 2021;14(2):e006799. [doi: 10.1161/circheartfailure.119.006799]
5. National Pharmaceutical Regulatory Agency, Ministry of Health Malaysia. URL: https://npra.gov.my/index.php/en/ [accessed 2021-02-22]
6. García CH, Pinheiro L, Maciá MA, Stroe R, Georgescu A, Dondera R, et al. Spontaneous adverse drug reactions: subgroup report. Heads of Medicines Agencies, European Medicines Agency. URL: https://www.ema.europa.eu/en/documents/report/spontaneous-adverse-drug-reactions-subgroup-report_en.pdf [accessed 2021-05-22]
7. Bergvall T, Norén GN, Lindquist M. vigiGrade: a tool to identify well-documented individual case reports and highlight systematic data quality issues. Drug Saf. Dec 17, 2013;37(1):65-77. [doi: 10.1007/s40264-013-0131-x]
