# Supplementary material for: Data-Driven Identification of Factors That Influence the Quality of Adverse Event Reports: 15-Year Interpretable Machine Learning and Time-Series Analyses of VigiBase and QUEST
Source: JMIR Med Inform. 2024 Apr 3;12:e49643. doi: 10.2196/49643 (PMC11024759; doi:10.2196/49643)
Supplement: Multimedia Appendix 2 [file medinform_v12i1e49643_app2.pdf]

Multimedia Appendix 2

Supplementary figures and tables

Figure S1. Spontaneous reporting mechanism in Malaysia.

Figure S2. Flowchart of applied report filters for Malaysian reports.

Figure S3. Top ranked features associated with well-documented reports from RF models.

Figure S4. Distribution of proportion of well-documented reports by reporter qualification and means of reporting since QUEST3+ officially launched in 2017.

Figure S5. Heatmap of average completeness of individual dimensions for complete E2B data set and selected E2B subsets during 2015-2019.

Table S1. Summary of report characteristics by reporting format and well-documented status

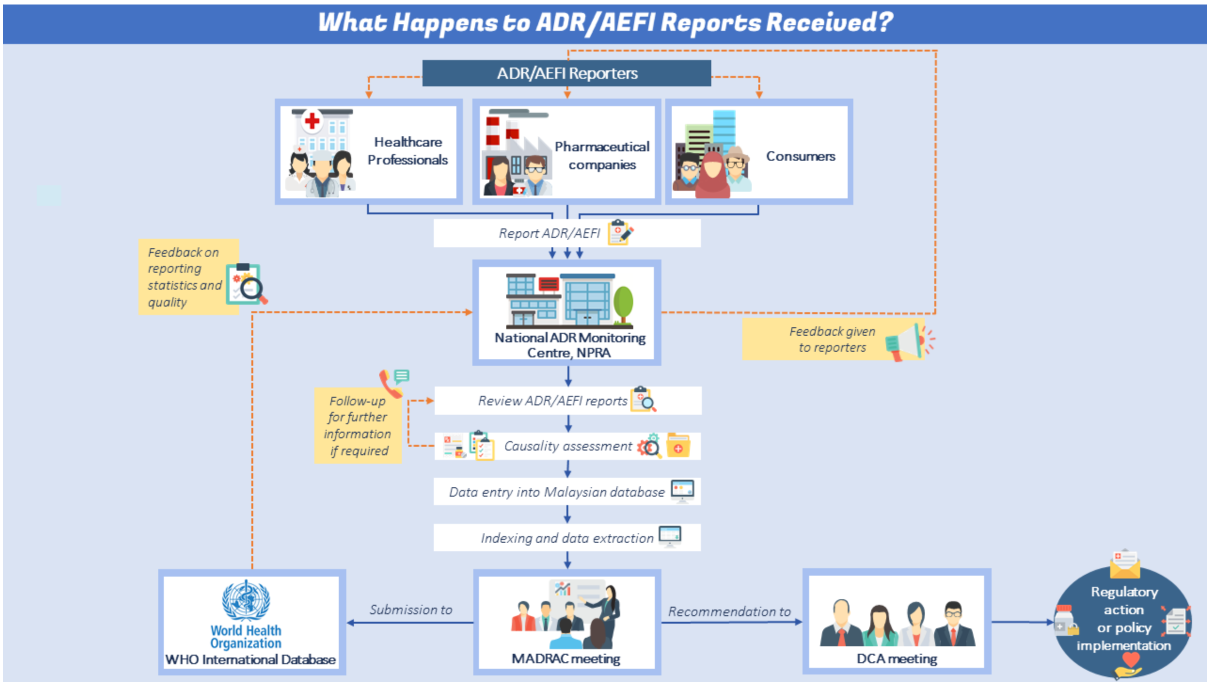

Figure S1. Spontaneous reporting mechanism in Malaysia [1].

Reference:

1. National Pharmaceutical Regulatory Agency, Ministry of Health Malaysia. URL: https://npra.gov.my/index.php/en/ [accessed 2021-02-22]

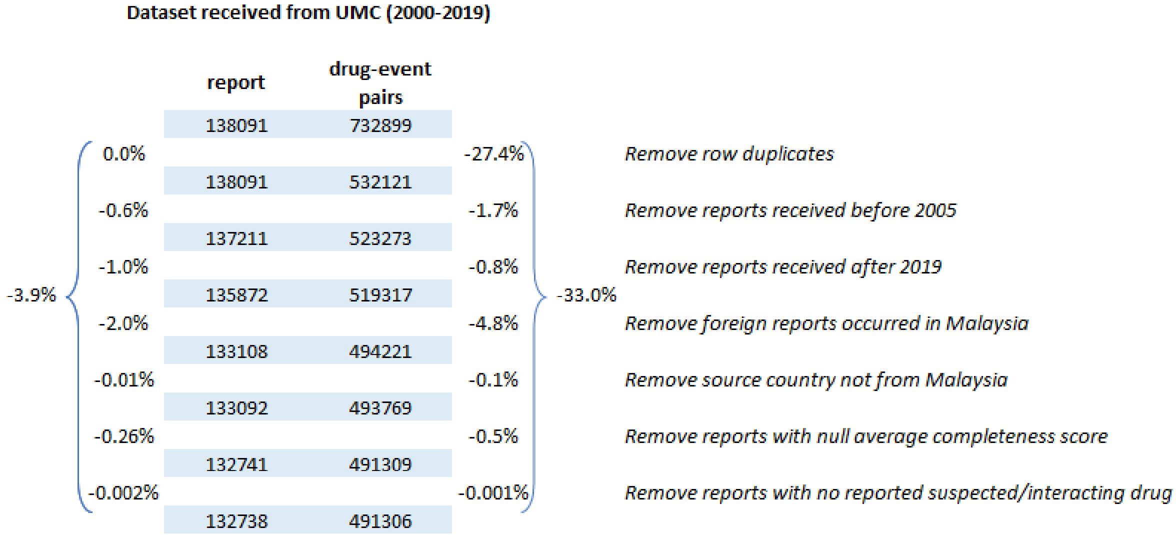

Figure S2. Flowchart of applied report filters for Malaysian reports.

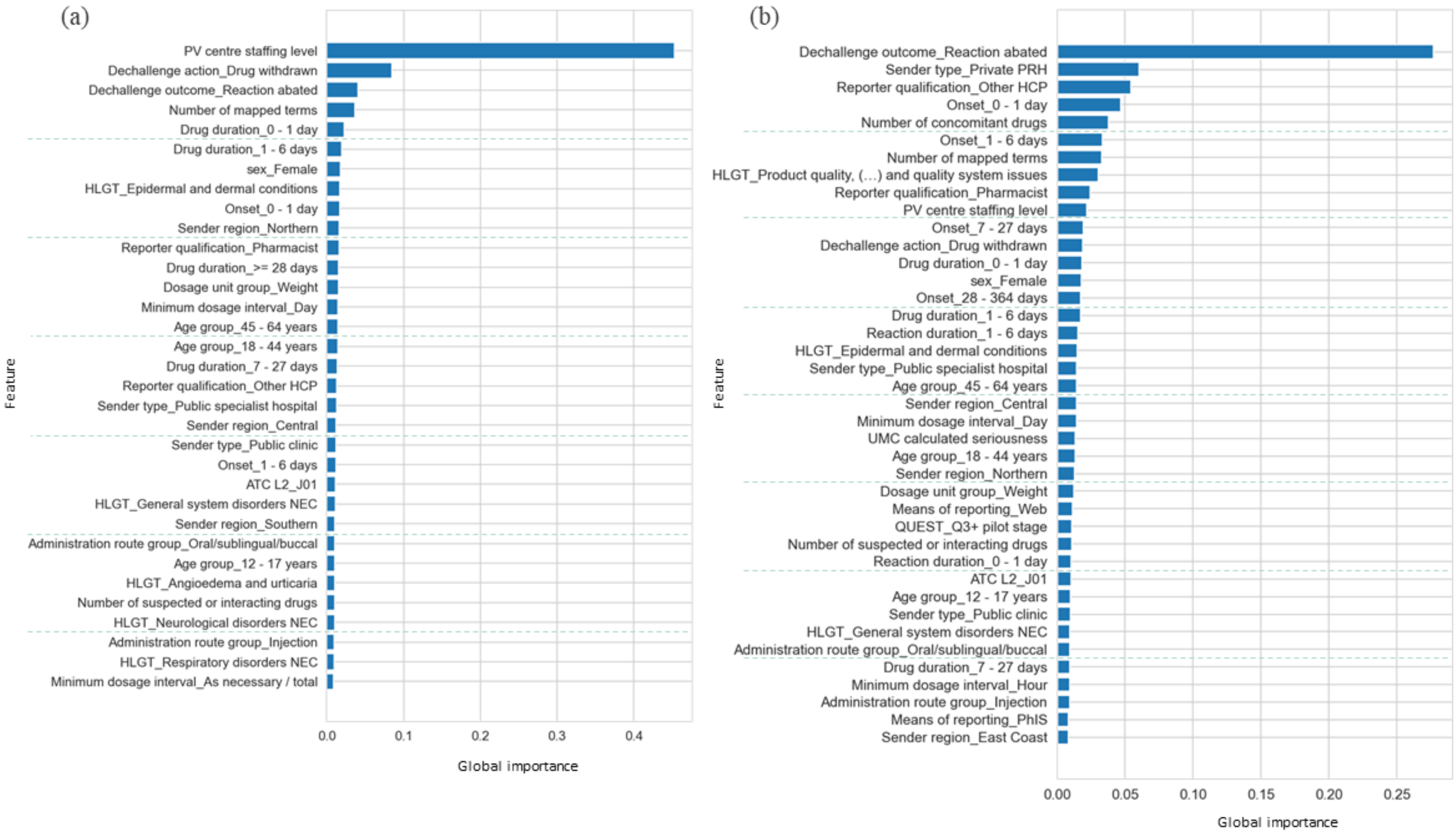

Figure S3. Top Important features associated with well-documented reports from RF models. **(a)** INTDIS subset: 33 features; **(b)** E2B subset: 40 features. Features are ordered according to their global importance. HCP: health care professional; HLGT: (MedDRA) High-Level Group Terms; PhIS: pharmacy hospital information system; PRH: product registration holder; PV: pharmacovigilance; UMC calculated seriousness: serious cases classified automatically by a UMC-developed algorithm.

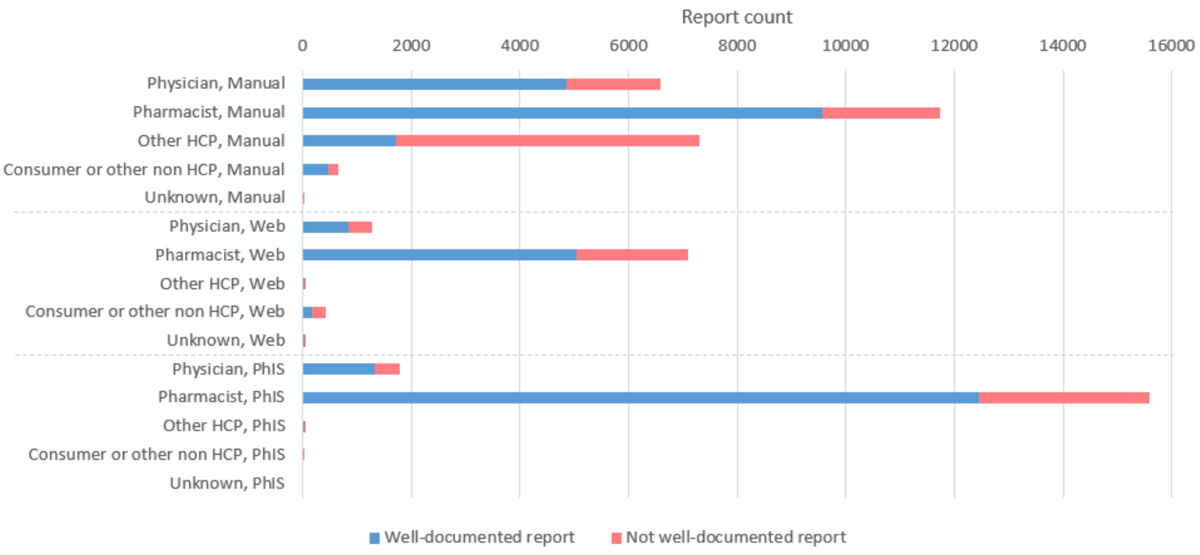

Figure S4. Distribution of proportion of well-documented reports by reporter qualification and means of reporting since QUEST3+ officially launched in 2017. HCP: health care professional; PhIS: pharmacy hospital information system; PRH: product registration holder.

	Overall	Onset	Outcome	Indication	Dosage	Sex	Age	Qualifi-cation	Report Type	Free Text
**Overall**	**0.80**	**0.84**	**0.82**	**0.94**	**0.41**	**0.97**	**0.95**	**0.98**	**1.00**	**0.99**
**Report type**										
Spontaneous	0.81	0.86	0.83	0.94	0.43	0.97	0.95	0.98	1.00	0.99
Report from study	0.44	0.27	0.43	0.94	0.01	0.90	0.83	0.99	1.00	0.99
Other	0.48	0.38	0.60	0.77	0.10	1.00	0.80	0.90	1.00	0.90
Not available to sender (unknown)	0.50	0.52	0.61	0.79	0.22	0.84	0.75	0.95	0.00	0.95
(blank)	0.73	0.82	0.88	1.00	0.34	1.00	0.96	0.91	0.00	1.00
**Reporting tool**										
Manual	0.77	0.81	0.78	0.91	0.41	0.95	0.92	0.97	1.00	0.99
PhIS	0.88	0.95	0.86	0.98	0.44	0.99	1.00	1.00	1.00	1.00
Web	0.80	0.79	0.89	0.98	0.35	1.00	0.97	0.95	0.99	1.00
**Sender type**										
Public specialist hospital	0.87	0.93	0.92	0.94	0.46	0.99	1.00	0.98	1.00	1.00
Public non-specialist hospital	0.87	0.93	0.90	0.96	0.45	0.99	1.00	0.98	1.00	1.00
University hospital	0.86	0.95	0.90	0.88	0.43	0.99	0.99	0.99	1.00	1.00
Public clinic	0.82	0.90	0.79	0.93	0.46	0.99	0.99	0.98	1.00	0.99
Other public services	0.65	0.75	0.50	0.50	0.50	1.00	1.00	1.00	1.00	1.00
Private PRH	0.39	0.25	0.36	0.94	0.05	0.76	0.61	0.99	1.00	0.99
Private hospital/clinic	0.84	0.91	0.90	0.92	0.38	0.99	0.98	0.97	1.00	0.99
Private community pharmacy	0.57	0.52	0.53	0.95	0.21	0.78	0.76	0.99	1.00	0.98
Consumer	0.80	0.92	0.69	0.96	0.53	0.96	0.94	0.98	1.00	1.00
(blank)	0.76	0.79	0.83	0.94	0.40	0.97	0.89	0.83	0.99	0.99
**Sender region**										
Central	0.69	0.69	0.70	0.94	0.31	0.91	0.86	0.98	1.00	0.99
Northern	0.86	0.93	0.88	0.93	0.46	0.99	0.99	0.98	1.00	0.99
Southern	0.84	0.91	0.87	0.92	0.47	0.99	0.99	0.98	1.00	0.99
East Malaysia	0.86	0.94	0.87	0.94	0.47	0.99	0.99	0.98	1.00	1.00
East Coast	0.86	0.91	0.89	0.96	0.44	0.99	0.99	0.98	1.00	0.99
(blank)	0.76	0.80	0.81	0.94	0.40	0.97	0.89	0.84	0.99	0.99
**Reporter qualification**										
Physician	0.84	0.90	0.84	0.94	0.44	0.99	0.99	1.00	1.00	0.99
Pharmacist	0.87	0.93	0.88	0.95	0.45	0.99	0.99	1.00	1.00	1.00
Other HCP	0.49	0.43	0.49	0.86	0.22	0.82	0.71	1.00	1.00	0.98
Consumer or other non-HCP	0.82	0.92	0.77	0.90	0.52	0.97	0.96	1.00	0.99	0.98
(blank)	0.71	0.82	0.85	0.94	0.41	0.98	0.91	0.00	1.00	0.99
**UMC calculated serious**										
Serious	0.77	0.80	0.83	0.92	0.38	0.95	0.93	0.98	1.00	1.00
Non-serious	0.81	0.85	0.81	0.94	0.42	0.97	0.95	0.98	1.00	0.99
**UMC calculated fatal**										
Fatal	0.55	0.38	0.92	0.91	0.10	0.85	0.76	0.99	1.00	1.00
Non-fatal	0.80	0.85	0.81	0.94	0.42	0.97	0.95	0.98	1.00	0.99

Figure S5. Heatmap of average completeness of individual dimensions for complete E2B data set and selected E2B subsets during 2015-2019. HCP: health care professional; PRH: product registration holder; UMC calculated seriousness: serious cases classified automatically by a UMC-developed algorithm. UMC calculated fatal: cases classified automatically by a UMC-developed algorithm.

Table S1. Summary of report characteristics by reporting format and well-documented status.

			INTDIS				E2B			
			63943		48.2%		68795		51.8%	
	Grand Total		No		Yes		No		Yes	
	**132738**		**53252**	83.3%	**10691**	16.7%	**22609**	32.9%	**46186**	67.1%
**Administrative**										
**No. of PV centre staff, annually, mean (SD)**	18	±6.7	11.2	±6.3	20.0	±2.2	22.2	±0.9	22.4	±0.8
**QUEST system**										
QUEST2	63943	48.2%	53252	100.0%	10691	100.0%				
QUEST3	16126	12.1%					6541	28.9%	9585	20.8%
QUEST3+	52669	39.7%					16068	71.1%	36601	79.2%
**Reporting tool**										
Manual	42437	32.0%					16200	71.7%	26237	56.8%
PhIS	17439	13.1%					3607	16.0%	13832	29.9%
Web	8919	6.7%					2802	12.4%	6117	13.2%
**Report type**										
Spontaneous	129872	97.8%	53086	99.7%	10689	100.0%	20282	89.7%	45815	99.2%
Report from study	2629	2.0%	53	0.1%	2	0.0%	2263	10.0%	311	0.7%
PMS/Special monitoring	60	0.0%	60	0.1%	0		0	0.0%	0	0.0%
Not available to sender (unknown)	44	0.0%					32	0.1%	12	0.0%
Other	10	0.0%					8	0.0%	2	0.0%
(blank)	123	0.1%	53	0.1%	0		24	0.1%	46	0.1%
Manual/web	63943	48.2%	53252	100.0%	10691	100.0%				
**Sender**										
**Sender type**										
Public specialist hospital	63064	47.5%	25208	47.3%	6022	56.3%	6353	28.1%	25481	55.2%
Public clinic	37473	28.2%	15239	28.6%	3232	30.2%	6625	29.3%	12377	26.8%
Private PRH	14459	10.9%	6465	12.1%	161	1.5%	7055	31.2%	778	1.7%
Public non-specialist hospital	8673	6.5%	2663	5.0%	738	6.9%	1056	4.7%	4216	9.1%
Private hospital/clinic	4357	3.3%	1625	3.1%	343	3.2%	618	2.7%	1771	3.8%
University hospital	2039	1.5%	1414	2.7%	117	1.1%	129	0.6%	379	0.8%
Other public service	369	0.3%	364	0.7%	1	0.0%	2	0.0%	2	0.0%
Private community pharmacy	139	0.1%	16	0.0%	3	0.0%	78	0.3%	42	0.1%
Consumer	52	0.0%	0		0		22	0.1%	30	0.1%
(blank)	2113	1.6%	258	0.5%	74	0.7%	671	3.0%	1110	2.4%
**Sender region**										
Central, West Malaysia	42090	31.7%	16898	31.7%	2898	27.1%	10522	46.5%	11772	25.5%
Northern, West Malaysia	32577	24.5%	14895	28.0%	2530	23.7%	3804	16.8%	11348	24.6%
Southern, West Malaysia	21391	16.1%	8190	15.4%	2177	20.4%	3196	14.1%	7828	16.9%
East Malaysia	17813	13.4%	7246	13.6%	1630	15.2%	2180	9.6%	6757	14.6%
East Coast, West Malaysia	16635	12.5%	5759	10.8%	1382	12.9%	2183	9.7%	7311	15.8%
(blank)	2232	1.7%	264	0.5%	74	0.7%	724	3.2%	1170	2.5%
**Reporter**										
**Reporter qualification**										
Pharmacist	75921	57.2%	26045	48.9%	6736	63.0%	9581	42.4%	33559	72.7%
Other	28141	21.2%	15573	29.2%	1890	17.7%	8484	37.5%	2194	4.8%
Physician	26848	20.2%	11559	21.7%	2051	19.2%	3843	17.0%	9395	20.3%
Consumer or other non health professional	150	0.1%	25	0.0%	1	0.0%	47	0.2%	77	0.2%
(blank)	1678	1.3%	50	0.1%	13	0.1%	654	2.9%	961	2.1%
**Patient**										
**Sex**										
Female	76833	57.9%	32058	60.2%	6310	59.0%	11897	52.6%	26568	57.5%
Male	51716	39.0%	19389	36.4%	4381	41.0%	8328	36.8%	19618	42.5%
(blank)	4189	3.2%	1805	3.4%	0	0.0%	2384	10.5%	0	
**Age group**										
0–27 days	218	0.2%	131	0.2%	21	0.2%	17	0.1%	49	0.1%
28 days–23 months	4876	3.7%	1263	2.4%	432	4.0%	669	3.0%	2512	5.4%
2–11 years	7752	5.8%	2124	4.0%	589	5.5%	1079	4.8%	3960	8.6%
12–17 years	13223	10.0%	7724	14.5%	1167	10.9%	1744	7.7%	2588	5.6%
18–44 years	38998	29.4%	14426	27.1%	3254	30.4%	5214	23.1%	16104	34.9%
45–64 years	40292	30.4%	16304	30.6%	3504	32.8%	6399	28.3%	14085	30.5%
65–74 years	13819	10.4%	5340	10.0%	1190	11.1%	2520	11.1%	4769	10.3%
≥75 years	6050	4.6%	2277	4.3%	534	5.0%	1157	5.1%	2082	4.5%
Unknown	7510	5.7%	3663	6.9%	0	0.0%	3810	16.9%	37	0.1%

			INTDIS				E2B			
			63943		48.2%		68795		51.8%	
	Grand Total		No		Yes		No		Yes	
**Case-level involvement (Presence)**	**132738**		**53252**	83.3%	**10691**	16.7%	**22609**	32.9%	**46186**	67.1%
**Drug**										
**No. of suspected or interacting drugs, mean ± SD**	1.10	±0.4	1.10	±0.39	1.08	±0.33	1.13	±0.54	1.09	±0.38
**No. of concomitant drugs, mean ± SD**	0.95	±1.7	0.87	±1.44	1.16	±1.57	0.82	±1.84	1.07	±2.02
**Top reported ATC Level 2***										
J01 Antibacterials for systemic use	26961	20.3%	8021	15.1%	2460	23.0%	2985	13.2%	13495	29.2%
J07 Vaccines	14612	11.0%	9108	17.1%	1184	11.1%	1992	8.8%	2328	5.0%
M01 Anti-inflammatory and antirheumatic products	11222	8.5%	3153	5.9%	1012	9.5%	1365	6.0%	5692	12.3%
C09 Agents acting on the renin–angiotensin system	8512	6.4%	3323	6.2%	626	5.9%	1876	8.3%	2687	5.8%
C08 Calcium channel blockers	7668	5.8%	2520	4.7%	735	6.9%	1910	8.4%	2503	5.4%
L01 Antineoplastic agents	6800	5.1%	2672	5.0%	288	2.7%	2757	12.2%	1083	2.3%
N02 Analgesics	5996	4.5%	2120	4.0%	281	2.6%	763	3.4%	2832	6.1%
A10 Drugs used in diabetes	5116	3.9%	1982	3.7%	475	4.4%	1125	5.0%	1534	3.3%
C10 Lipid modifying agents	5225	3.9%	2365	4.4%	444	4.2%	736	3.3%	1680	3.6%
B01 Antithrombotic agents	3726	2.8%	1562	2.9%	516	4.8%	546	2.4%	1102	2.4%
**Administration route group***										
Oral/sublingual/buccal	83754	63.1%	31593	59.3%	6740	63.0%	14562	64.4%	30859	66.8%
Injection	36388	27.4%	16033	30.1%	3515	32.9%	4610	20.4%	12230	26.5%
Topical	1912	1.4%	875	1.6%	74	0.7%	455	2.0%	508	1.1%
Others	1564	1.2%	510	1.0%	68	0.6%	272	1.2%	714	1.5%
(blank)	10335	7.8%	5149	9.7%	452	4.2%	2374	10.5%	2360	5.1%
Unknown	1241	0.9%					775	3.4%	466	1.0%
**Dosage unit group***										
Weight	92586	69.8%	35491	66.6%	8058	75.4%	16292	72.1%	32745	70.9%
Volume	21068	15.9%	8280	15.5%	1271	11.9%	3744	16.6%	7773	16.8%
Dosage form	14058	10.6%	4450	8.4%	897	8.4%	2813	12.4%	5898	12.8%
Standardised unit	3731	2.8%	1157	2.2%	338	3.2%	717	3.2%	1519	3.3%
Others	104	0.1%	28	0.1%	7	0.1%	23	0.1%	46	0.1%
(blank)	11216	8.4%	4684	8.8%	251	2.3%	2059	9.1%	4222	9.1%
**Minimum dosing interval unit***										
As necessary/total	18930	14.3%	11947	22.4%	3106	29.1%	1659	7.3%	2218	4.8%
Hour	20313	15.3%					4289	19.0%	16024	34.7%
Day	70380	53.0%	31615	59.4%	6750	63.1%	9799	43.3%	22216	48.1%
Week	1029	0.8%	450	0.8%	39	0.4%	284	1.3%	256	0.6%
Month	368	0.3%	179	0.3%	18	0.2%	104	0.5%	67	0.1%
Year	15	0.0%	12	0.0%	0	0.0%	2	0.0%	1	0.0%
Cyclical	15	0.0%	11	0.0%	4	0.0%				
(blank)	23881	18.0%	9754	18.3%	883	8.3%	6995	30.9%	6249	13.5%
**Drug duration group***										
<1 day	36309	27.4%	11607	21.8%	4300	40.2%	3779	16.7%	16623	36.0%
1–6 days	28772	21.7%	10282	19.3%	3390	31.7%	2382	10.5%	12718	27.5%
7–27 days	9305	7.0%	3684	6.9%	1142	10.7%	1044	4.6%	3435	7.4%
≥28 days	11982	9.0%	5175	9.7%	1312	12.3%	1846	8.2%	3649	7.9%
(blank)	48589	36.6%	23609	44.3%	735	6.9%	13887	61.4%	10358	22.4%
**Dechallenge action***										
Drug withdrawn	95474	71.9%	36205	68.0%	10461	97.8%	12676	56.1%	36132	78.2%
Not applicable	10003	7.5%					3239	14.3%	6764	14.6%
Dose not changed	2727	2.1%	950	1.8%	107	1.0%	915	4.0%	755	1.6%
Dose reduced	794	0.6%					410	1.8%	384	0.8%
Dose increased	132	0.1%					111	0.5%	21	0.0%
Unknown	24392	18.4%	16091	30.2%	123	1.2%	5659	25.0%	2519	5.5%
(blank)	159	0.1%	6	0.0%	0	0.0%	64	0.3%	89	0.2%
**Rechallenge action***										
Rechallenge	71219	53.7%	2329	4.4%	169	1.6%	22580	99.9%	46141	99.9%
No rechallenge	58824	44.3%	48323	90.7%	10501	98.2%				
Unknown	2613	2.0%	2592	4.9%	21	0.2%				
(blank)	509	0.4%	9	0.0%	0	0.0%	156	0.7%	344	0.7%

*Only observations related to suspected/interacting drug

	Grand Total		INTDIS				E2B			
			63943		48.2%		68795		51.8%	
			No		Yes		No		Yes	
**Case-level involvement (Presence)**	**132738**		**53252**	83.3%	**10691**	16.7%	**22609**	32.9%	**46186**	67.1%
**Reaction**										
**No. of mapped terms, mean (SD)**	1.80	±1.1	1.8	±1.1	1.8	±1.0	1.8	±1.2	1.8	±1.1
**Top reported HLGT***										
Epidermal and dermal conditions	43249	32.6%	14207	26.7%	3813	35.7%	5521	24.4%	19708	42.7%
General system disorders NEC	18155	13.7%	7566	14.2%	1382	12.9%	2750	12.2%	6457	14.0%
Neurological disorders NEC	15116	11.4%	7027	13.2%	1274	11.9%	2554	11.3%	4261	9.2%
Respiratory disorders NEC	12684	9.6%	4513	8.5%	1034	9.7%	2036	9.0%	5101	11.0%
Angioedema and urticaria	12173	9.2%	2897	5.4%	1417	13.3%	1340	5.9%	6519	14.1%
Gastrointestinal signs and symptoms	11269	8.5%	5433	10.2%	847	7.9%	1853	8.2%	3136	6.8%
Administration site reactions	11226	8.5%	7019	13.2%	905	8.5%	1364	6.0%	1938	4.2%
Eye disorders NEC	9046	6.8%	1868	3.5%	606	5.7%	1079	4.8%	5493	11.9%
Headaches	6812	5.1%	3602	6.8%	518	4.8%	1033	4.6%	1659	3.6%
Gastrointestinal motility and defaecation conditions	4280	3.2%	1512	2.8%	247	2.3%	660	2.9%	1861	4.0%
Oral soft tissue conditions	3469	2.6%	1176	2.2%	283	2.6%	432	1.9%	1578	3.4%
Product quality, supply, distribution, manufacturing and quality system issues	3264	2.5%	365	0.7%	292	2.7%	1843	8.2%	764	1.7%
**Time-to-onset***										
<1 day	63797	48.1%	22258	41.8%	6287	58.8%	6348	28.1%	28904	62.6%
1–6 days	30955	23.3%	13400	25.2%	2632	24.6%	3457	15.3%	11466	24.8%
7– 27 days	10366	7.8%	4221	7.9%	940	8.8%	1409	6.2%	3796	8.2%
28–364 days	7349	5.5%	3132	5.9%	623	5.8%	1408	6.2%	2186	4.7%
≥365 days	2186	1.6%	1134	2.1%	182	1.7%	419	1.9%	451	1.0%
**Reaction duration group***										
<1 day	11423	8.6%					1630	7.2%	9793	21.2%
1–6 days	13243	10.0%					1558	6.9%	11685	25.3%
7–27 days	2779	2.1%					463	2.0%	2316	5.0%
≥28 days	1693	1.3%					397	1.8%	1296	2.8%
(blank)	105712	79.6%	53252	100.0%	10691	100.0%	19352	85.6%	22417	48.5%
**Dechallenge outcome**										
Reaction abated	93027	70.1%	28170	52.9%	8694	81.3%	10297	45.5%	45866	99.3%
No effect observed	9855	7.4%	8035	15.1%	1767	16.5%	19	0.1%	34	0.1%
Fatal	1010	0.8%		0.0%		0.0%	717	3.2%	293	0.6%
Effect unknown	12154	9.2%		0.0%		0.0%	12059	53.3%	95	0.2%
(blank)	2712	2.0%	6	0.0%	0	0.0%	2022	8.9%	684	1.5%
Not applicable	17271	13.0%	17041	32.0%	230	2.2%				
**Rechallenge outcome**										
Effect unknown	70796	53.3%	2592	4.9%	21	0.2%	22461	99.3%	45722	99.0%
Reaction recurred	2775	2.1%	2124	4.0%	135	1.3%	91	0.4%	425	0.9%
No recurrence	342	0.3%	205	0.4%	34	0.3%	44	0.2%	59	0.1%
Not applicable	58824	44.3%	48323	90.7%	10501	98.2%		0.0%		0.0%
(blank)	509	0.4%	9	0.0%	0	0.0%	156	0.7%	344	0.7%
**UMC calculated seriousness**										
Yes	14666	11.0%	868	1.6%	106	1.0%	4946	21.9%	8746	18.9%
**UMC calculated fatal**										
Yes	2022	1.5%	868	1.6%	106	1.0%	750	3.3%	298	0.6%

*Only observations related to suspected/interacting drug

Mapped term: MedDRA Preferred Term (PT); ATC: Anatomical Therapeutic Code; HCP: health care professional; HLGT: (MedDRA) High-Level Group Terms; NEC: not elsewhere classified; PhIS: Pharmacy hospital information system; PMS: post-marketing surveillance; PRH: product registration holder; PV: pharmacovigilance; UMC calculated seriousness: serious cases classified automatically by a UMC-developed algorithm; UMC calculated fatal: cases classified automatically by a UMC-developed algorithm.
