# Supplementary material for: Data-Driven Identification of Factors That Influence the Quality of Adverse Event Reports: 15-Year Interpretable Machine Learning and Time-Series Analyses of VigiBase and QUEST
Source: JMIR Med Inform. 2024 Apr 3;12:e49643. doi: 10.2196/49643 (PMC11024759; doi:10.2196/49643)
Supplement: Multimedia Appendix 3 [file medinform_v12i1e49643_app3.pdf]

Multimedia Appendix 3

Literature review on quality of reports in spontaneous reporting system

We searched MEDLINE database for recent studies on spontaneous reporting system (SRS) data quality between 2014 and February 2021. The following search terms were used: ("spontaneous report*" OR "adverse drug reaction report*" OR "ADR report*" OR "adverse event report*" OR "AE report*" OR "Individual Case Safety Report" OR "ICSR" OR "pharmacovigilance") AND ("quality" OR "completeness"). The search was limited to English language studies and full-text availability. Of the 6,257 studies, abstracts were screened for relevance to the topic. Furthermore, a reference list search and a cited reference search were carried out based on full-text papers meeting the study selection criteria. Studies that reported only completeness score or well-documented rate without discussing factors that may contribute were excluded. As a result, 25 studies were reviewed in total.

Among the 25 studies reviewed, 18 examined reports recorded in the national or regional databases [1-18], six evaluated VigiBase reports [19-24], and one studied the global safety databases of pharmaceutical companies [25]. While most were descriptive cross-sectional studies, there were two quasi-experimental studies that evaluated the impact of pharmacovigilance assessors [1] and the new consumer reporting form in plain language [2]. Most studies employed a single method to evaluate report quality, whereas some used multiple — vigiGrade was used in six studies [19-24] and adapted in seven [3-8, 25], clinical documentation tool (ClinDoc) in three [2, 9, 24], WHO documentation grading in two [7, 10], while the other studies employed different scoring methods. It is, however, important to note that studies applying vigiGrade in different data sources other than VigiBase may have found different completeness score, as they did not take into account for possible transmission errors [19] or the study datasets may not have contained all granularity of E2B format [26] to reproduce the multiplicative scoring method. Compared to studies that evaluated reporting

rates, the significant methodological heterogeneity of studies on reporting quality makes comparing their overall completeness difficult.

Diverse findings pertaining to factors associated with report completeness have been observed among different countries and stakeholders, depending on the existence of regulations, practices, resources, and cultures of reporting. Factors frequently reported were included sender type (e.g., medical institutions versus pharmaceutical companies) [4-6, 10-15, 19, 21], reporter qualification [6, 8, 10, 13-15, 18, 19, 21, 23], means of reporting (e.g., app reporting, plain-language forms) [2, 3, 12, 19, 21, 24], case seriousness or outcome [12-14, 16, 17], report type (e.g., study reports, program-specific reports) [7, 22, 25], patient age group [15, 17], patient sex [15], and causality [15]. Importantly, most included studies performed simple univariable analysis to examine the relationships among subcategories of a single variable and report completeness, whilst three have used multivariable logistic regression to study factors associated with well-documented reports [15, 16] or completeness of specific information [18]. Probably owing to the complex data structure in E2B format (as one report may contain multiple drugs and reactions), Toki and Ono [18] assessed only factors related to primary suspect drugs. Another two multivariable models did not study the relationships between characteristics of suspect drugs or reactions and report quality [15, 16].

References

1. Kheloufi F, Default A, Rouby F, Laugier-Castellan D, Boyer M, Rodrigues B, et al. Informativeness of patient initial reports of adverse drug reactions. Can it be improved by a pharmacovigilance centre? Eur J Clin Pharmacol. Aug 2017;73(8):1009-1018. [doi: 10.1007/s00228-017-2254-y] [Medline: 28391408]
2. Muñoz MA, Delcher C, Dal Pan GJ, Kortepeter CM, Wu E, Wei YJ, et al. Impact of a new consumer form on the quantity and quality of adverse event reports submitted to the United States Food and Drug Administration. Pharmacotherapy. Nov 2019;39(11):1042-1052. [doi: 10.1002/phar.2325] [Medline: 31479525]
3. Bahk CY, Goshgarian M, Donahue K, Freifeld CC, Menone CM, Pierce CE, et al. Increasing patient engagement in pharmacovigilance through online community outreach and mobile reporting applications: an analysis of adverse event reporting for the Essure

device in the US. Pharmaceut Med. 2015;29(6):331-340. [FREE Full text] [doi:10.1007/s40290-015-0106-6] [Medline: 26635479]

4. Plessis L, Gómez A, García N, Cereza G, Figueras A. Lack of essential information in spontaneous reports of adverse drug reactions in Catalonia—a restraint to the potentiality for signal detection. Eur J Clin Pharmacol. Mar 1, 2017;73(6):751-758. [doi: 10.1007/s00228-017-2223-5]
5. Fernandez-Fernandez C, Lázaro-Bengoa E, Fernández-Antón E, Quiroga-González L, Montero Corominas D. Quantity is not enough: completeness of suspected adverse drug reaction reports in Spain-differences between regional pharmacovigilance centres and pharmaceutical industry. Eur J Clin Pharmacol. Aug 2020;76(8):1175-1181. [doi: 10.1007/s00228-020-02894-0] [Medline: 32447435]
6. Tsuchiya M, Obara T, Miyazaki M, Noda A, Takamura C, Mano N. The quality assessment of the Japanese Adverse Drug Event Report database using vigiGrade. Int J Clin Pharm. Apr 2020;42(2):728-736. [doi: 10.1007/s11096-020-00969-7] [Medline: 32020439]
7. Tsuchiya M, Obara T, Miyazaki M, Noda A, Sakai T, Funakoshi R, et al. High-quality reports and their characteristics in the Japanese Adverse Drug Event Report database (JADER). J Pharm Pharm Sci. Apr 08, 2021;24:161-173. [doi:10.18433/jpps31417]
8. Araujo AG, Lucchetta RC, Tonin FS, Pontarolo R, Borba HH, Wiens A. Analysis of completeness for spontaneous reporting of disease-modifying therapies in multiple sclerosis. Expert Opin Drug Saf. Mar 11, 2021;20(6):735-740. [doi: 10.1080/14740338.2021.1897566]
9. Rolfes L, van Hunsel F, van der Linden L, Taxis K, van Puijenbroek E. The quality of clinical information in adverse drug reaction reports by patients and healthcare professionals: a retrospective comparative analysis. Drug Saf. Jul 2017;40(7):607-614. [FREE Full text] [doi: 10.1007/s40264-017-0530-5] [Medline: 28405899]
10. Tsuchiya M, Obara T, Sakai T, Nomura K, Takamura C, Mano N. Quality evaluation of the Japanese Adverse Drug Event Report database (JADER). Pharmacoepidemiol Drug. Dec 10, 2019;29(2):173-181. [doi: 10.1002/pds.4944]
11. Ribeiro A, Lima S, Zampieri ME, Peinado M, Figueras A. Filling quality of the reports of adverse drug reactions received at the Pharmacovigilance Centre of São Paulo (Brazil): missing information hinders the analysis of suspected associations. Expert Opin Drug Saf. Aug 23, 2017;16(12):1329-1334. [doi: 10.1080/14740338.2017.1369525]
12. Moore TJ, Furberg CD, Mattison DR, Cohen MR. Completeness of serious adverse drug event reports received by the US Food and Drug Administration in 2014. Pharmacoepidemiol Drug Safety. Feb 10, 2016;25(6):713-718. [doi:10.1002/pds.3979]
13. Chen Y, Niu R, Xiang Y, Wang N, Bai J, Feng B. The quality of spontaneous adverse drug reaction reports in China: a descriptive study. Biol Pharml Bull. 2019;42(12):2083-2088. [doi: 10.1248/bpb.b19-00637]
14. Niu R, Xiang Y, Wu T, Zhang Z, Chen Y, Feng B. The quality of spontaneous adverse drug reaction reports from the pharmacovigilance centre in western China. Expert Opin Drug Saf. Jan 2019;18(1):51-58. [doi: 10.1080/14740338.2019.1559812] [Medline: 30574811]
15. Oh IS, Baek YH, Kim HJ, Lee M, Shin JY. Differential completeness of spontaneous adverse event reports among hospitals/clinics, pharmacies, consumers, and pharmaceutical

companies in South Korea. PLoS ONE. Feb 14, 2019;14(2):e0212336. [doi: 10.1371/journal.pone.0212336]

16. Durrieu G, Jacquot J, Mège M, Bondon-Guitton E, Rousseau V, Montastruc F, et al. Completeness of spontaneous adverse drug reaction reports sent by general practitioners to a regional pharmacovigilance centre: a descriptive study. Drug Saf. Sep 29, 2016;39(12):1189-1195. [doi: 10.1007/s40264-016-0463-4].
17. Humbert X, Jacquot J, Alexandre J, Sassier M, Robin N, Pageot C, et al. Completeness of pharmacovigilance reporting in general medicine in France. Sante Publique. 2019;31(4):561-566. [doi: 10.3917/spub.194.0561]
18. Toki T, Ono S. Assessment of factors associated with completeness of spontaneous adverse event reporting in the United States: a comparison between consumer reports and healthcare professional reports. J Clin Pharm Ther. Nov 25,2019;45(3):462-469. [doi: 10.1111/jcpt.13086]
19. Bergvall T, Norén GN, Lindquist M. vigiGrade: a tool to identify well-documented individual case reports and highlight systematic data quality issues. Drug Saf. Dec 17, 2013;37(1):65-77. [doi: 10.1007/s40264-013-0131-x]
20. Masuka JT, Khoza S. An analysis of the trends, characteristics, scope, and performance of the Zimbabwean pharmacovigilance reporting scheme. Pharmacol Res Perspect. Sep 15, 2020;8(5):e00657. [doi: 10.1002/prp2.657]
21. Thuy Nguyen Phuong ANH, Hoa Nguyen Dang. Developing quality in Vietnam. Uppsala reports 68. Uppsala Monitoring Centre. Jan 2015. URL: https://who-umc.org/media/164371/ur68_final_2_gb.pdf [accessed 2024-03-12]
22. Masuka JT, Khoza S. An analysis of the trends, characteristics, scope, and performance of the Zimbabwean pharmacovigilance reporting scheme. Pharmacol Res Perspect. Sep 15, 2020;8(5):e00657. [doi: 10.1002/prp2.657]
23. Wakao R, Taavola H, Sandberg L, Iwasa E, Soejima S, Chandler R, et al. Data-driven identification of adverse event reporting patterns for Japan in VigiBase, the WHO global database of individual case safety reports. Drug Saf. Sep 26,2019;42(12):1487-1498. [doi: 10.1007/s40264-019-00861-y]
24. Oosterhuis I, Taavola H, Tregunno PM, Mas P, Gama S, Newbould V, et al. Characteristics, quality and contribution to signal detection of spontaneous reports of adverse drug reactions via the WEB-RADR mobile application: a descriptive cross-sectional study. Drug Saf. Oct 2018;41(10):969-978. [FREE Full text] [doi: 10.1007/s40264-018-0679-6] [Medline: 29761281]
25. Jokinen J, Bertin D, Donzanti B, Hormbrey J, Simmons V, Li H, et al. Industry assessment of the contribution of patient support programs, market research programs, and social media to patient safety. Ther Innov Regul Sci. Nov 2019;53(6):736-745. [doi: 10.1177/2168479019877384] [Medline: 31684774]
26. Technical description of vigiGrade™: completeness score. Uppsala Monitoring Centre. URL: https://www.zva.gov.lv/archive/doc_upl/Technical-description-of-vigiGrade-Completeness-score.pdf [accessed 2024-03-11]
