# Supplementary material for: Data-Driven Identification of Factors That Influence the Quality of Adverse Event Reports: 15-Year Interpretable Machine Learning and Time-Series Analyses of VigiBase and QUEST
Source: JMIR Med Inform. 2024 Apr 3;12:e49643. doi: 10.2196/49643 (PMC11024759; doi:10.2196/49643)
Supplement: Multimedia Appendix 4 [file medinform_v12i1e49643_app4.pdf]

Multimedia Appendix 4

Feature selection

Introduction: Data-Driven Based Factor Selection

Data sparsity (variables dominated by zero values), multicollinearity, high computational cost, and model overfitting are issues typically accompanied by high dimensional health data, often referred to as the “curse of dimensionality” [1-3]. An overfitted model, characterized by much greater accuracy on observed data than unseen data, tends to capture noise or random errors rather than the underlying relationships between variables [4]. Multicollinearity does not reduce the model’s accuracy, but it adversely impacts their interpretability, or their capability to make reliable inferences about the individual variables. When the features (independent variables) in a model are highly correlated, the resulting inflated variance may dramatically impair the stability of effect size estimates (e.g., coefficients for regression models) or dilute the relative importance score of correlated features (e.g., for tree-based models) [5, 6].

Feature selection is a common technique for dealing with high dimensionality by removing redundant features. There is an overall trade-off between the benefits of reduced variance and the harm of increased bias, as narrowing feature sets may risk losing key mechanistic information. In fact, the effect of feature sets can be far greater than model types [2, 5, 6]. Traditional multivariable regression models use a hypothesis-driven framework to manually select variables based on domain knowledge and a literature review [7]. While this approach effectively narrows the study’s focus and minimizes false discoveries, selection bias may result in the exclusion of unanticipated factors associated with report quality. By allowing automated assessment of large numbers of report characteristics in a less human-biased manner, ML-based feature selection offers marked advantages in uncovering patterns that would have been overlooked using a hypothesis-based approach [7].

Over the years, various feature selection techniques have been developed in the ML domain and are generally grouped into filter, embedded, and wrapper methods (Figure 1). Filter methods evaluate the relevance of features using various statistical measures, and low-scoring features are removed. Most proposed filter methods are simple univariable filters that assess each feature independently, neglecting feature dependencies and interactions with the ML model. Wrapper methods iteratively select or eliminate subsets of features that work best for a given ML algorithm. Conversely, embedded methods have the intrinsic feature selection incorporated within the ML model fitting process [5, 8-10].

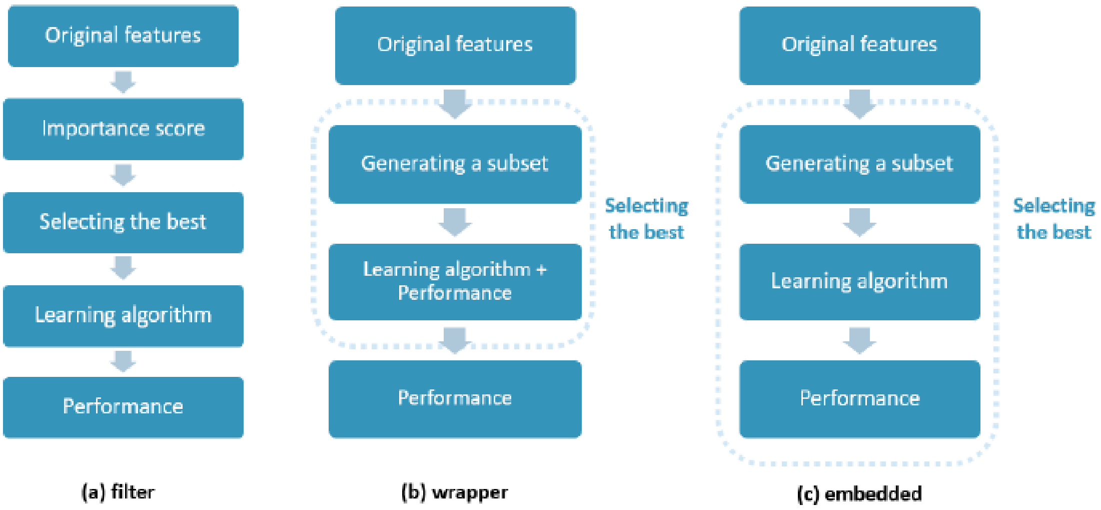

Figure 1: Types or feature selection methods.

Of these, wrapper methods have the advantage of finding the globally most important feature from a variety of feature subsets, but they are computationally expensive and risk overfitting with insufficient samples [5, 8, 10]. One of the most frequently used wrapper methods is recursive feature elimination (RFE). RFE is a backward selection approach that iteratively creates new models, removes the least important features one by one until all are explored, and ranks features based on the order of removal [5, 8]. As wrapper and embedded methods are ML algorithm dependent, certain algorithms, such as random forest (RF), benefit more from

RFE than others [5]. Moreover, modern tree-based feature selection methods, e.g., RF, achieve better parsimony in larger datasets for clinical predictive modelling than classical regression-based methods [11].

Recent studies have proposed a hybrid approach to pre-reduce the feature search space using a univariable filter method and subsequently employ the wrapper or embedded methods, therefore reducing overfitting risks and computation costs [5, 8]. Regardless, much like developing explanatory regression models, strict automation of feature selection is unlikely to be a realistic approach. In almost all domains, incorporating domain expertise into the data-driven feature selection process stands vital for developing a meaningful and effective model. [6].

Multivariable Machine Learning Analysis: Feature Selection Process

The detailed process for feature selection methods applied in the study is as follows:

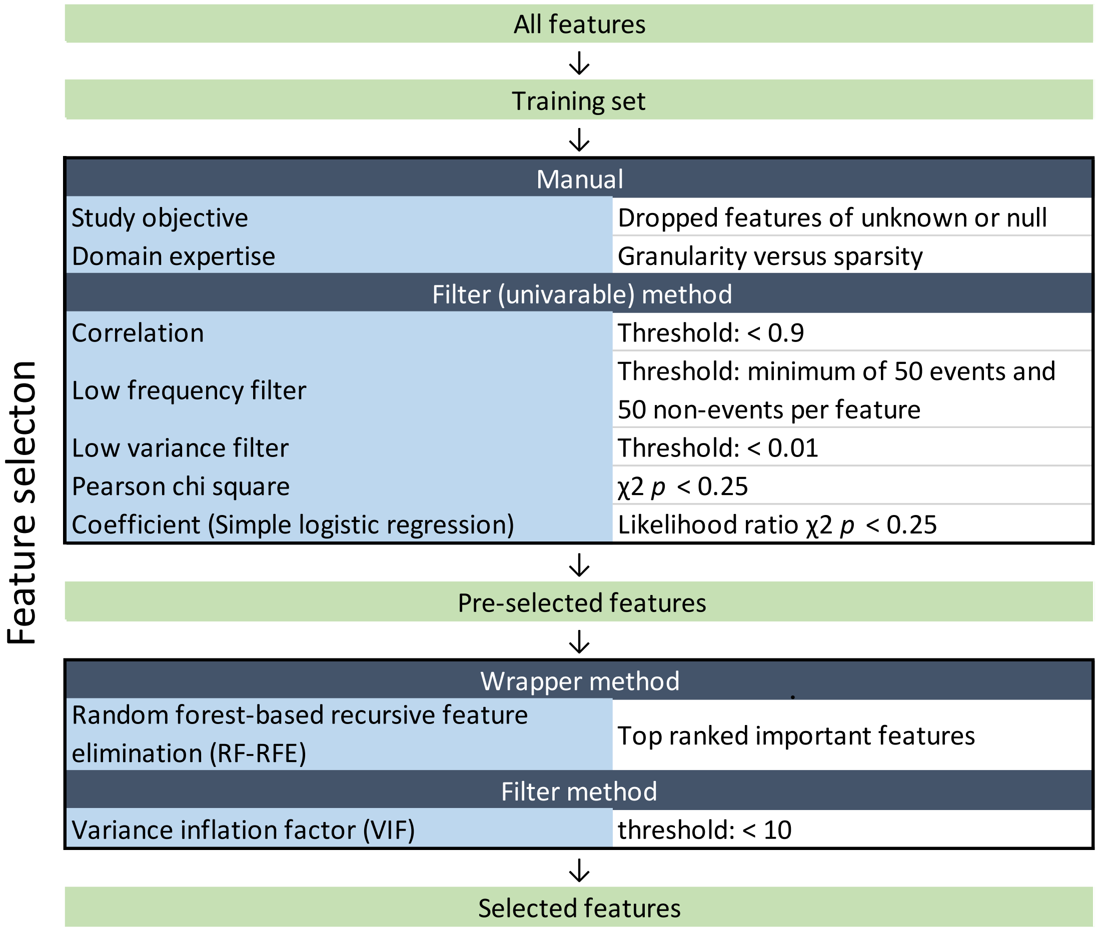

Figure 2: Flowchart of the two-stage feature selection process.

We performed a hybrid feature selection to eliminate redundant or less informative features before proceeding to data mining with the ML algorithm. To avoid the data leakage issue and the corresponding model overfitting, we conducted a two-stage feature selection solely based on training data [6, 12]. We first applied the univariable filter method to independently assess and pre-select the features, and subsequently selected the top-ranked features using a wrapper method coupled with an assessment of multicollinearity **(Figure 2)**. ***Manual/Filter method.*** We excluded features of the 'unknown' or 'null' category as they provided no additional insights in line with our study objectives. For structural multicollinearity (when one variable is

created from another), we considered a balance between granularity and sparsity while selecting a feature. For this reason, High-Level Group Terms (HLGT) and ATC level 2 subsets were chosen over other MedDRA and ATC hierarchical subsets. Highly correlated features [Pearson's R <0.9] were assessed and further compared over the strength of association with the outcome (whichever had a higher crude odds ratio and a higher Pearson's correlation with the outcome were retained). To ensure a stable model, features with near-zero variance (threshold=0.01, i.e., a single value in over 99% of the samples) and features with a lower expected count of 50 among events and non-events were excluded. Lastly, we retained only the features that indicated *p* values < .25 for Pearson's chi-square test (categorical features) and likelihood-ratio chi-square (all features) from simple logistic regression. ***Wrapper/Filter method.*** We first attempted the recursive feature elimination and cross-validation selection (RFECV, cv=5) algorithm with a random forest (RF) classifier to assess and visualize the best number of features for optimal model accuracy performance. We then used the RF-based recursive feature elimination (RF-RFE) method to rank the feature based on its importance score. The lowest ranked important variables were eliminated one by one until the final subset achieved a variance inflation factor (VIF) lower than 10 (a threshold for severe multicollinearity).

References

1. Altman N, Krzywinski M. The curse(s) of dimensionality. Nat Methods. Jun 2018;15(6):399-400. [doi:10.1038/s41592-018-0019-x] [Medline: 29855577]
2. Lee CH, Yoon HJ. Medical big data: promise and challenges. Kidney Res Clin Pract. Mar 31, 2017;36(1):3-11. [doi:10.23876/j.krcp.2017.36.1.3]
3. Stevens LM, Mortazavi BJ, Deo RC, Curtis L, Kao DP. Recommendations for reporting machine learning analyses in clinical research. Circ Cardiovasc Qual Outcomes. Oct 2020;13(10). [FREE Full text] [doi: 10.1161/circoutcomes.120.006556]
4. Lever J, Krzywinski M, Altman N. Model selection and overfitting. Nat Methods. Aug 30, 2016;13(9):703-704. [doi: 10.1038/nmeth.3968]
5. Kuhn M, Johnson K. Feature Engineering and Selection: A Practical Approach for Predictive Models. Boca Raton, FL. CRC Press; 2019. ISBN: 9781351609463.
6. Wiemken TL, Kelley RR. Machine learning in epidemiology and health outcomes research. Annu Rev Public Health. Apr 02, 2020;41:21-36. [FREE Full text] [doi: 10.1146/annurev-publhealth-040119-094437] [Medline: 31577910]
7. Stevens LM, Linstead E, Hall JL, Kao DP. Association between coffee intake and incident heart failure risk. Circ Heart Failure. Feb 2021;14(2):e006799. [doi: 10.1161/circheartfailure.119.006799]
8. Saeys Y, Inza I, Larrañaga P. A review of feature selection techniques in bioinformatics. Bioinformatics. Oct 01, 2007;23(19):2507-2517. [doi: 10.1093/bioinformatics/btm344] [Medline: 17720704]
9. Kohavi R, John GH. Wrappers for feature subset selection. Artif Intell. Dec 1997;97(1-2):273-324. [doi: 10.1016/S0004-3702(97)00043-X]
10. Guyon I, Elisseeff A. An Introduction of Variable and Feature Selection. J Machine Learning Research Special Issue on Variable and Feature Selection. 2003 01/01;3:1157-82. [doi: 10.1162/153244303322753616]
11. Sanchez-Pinto LN, Venable LR, Fahrenbach J, Churpek MM. Comparison of variable selection methods for clinical predictive modeling. Int J Med Inform. Aug 2018;116:10-17. [FREE Full text] [doi: 10.1016/j.ijmedinf.2018.05.006] [Medline: 29887230]
12. Luo W, Phung D, Tran T, Gupta S, Rana S, Karmakar C, et al. Guidelines for developing and reporting machine learning predictive models in biomedical research: a multidisciplinary view. J Med Internet Res. Dec 16, 2016;18(12):e323. [FREE Full text] [doi: 10.2196/jmir.5870]
