# Supplementary material for: Data-Driven Identification of Factors That Influence the Quality of Adverse Event Reports: 15-Year Interpretable Machine Learning and Time-Series Analyses of VigiBase and QUEST
Source: JMIR Med Inform. 2024 Apr 3;12:e49643. doi: 10.2196/49643 (PMC11024759; doi:10.2196/49643)
Supplement: Multimedia Appendix 5 [file medinform_v12i1e49643_app5.pdf]

Multimedia Appendix 5

Tree-based machine learning models and interpretable machine learning

Tree-based machine learning (ML) models are among the most popular ML methods and attain state-of-the-art performance in a wide variety of domains [1]. Tree-based models regularly outperform standard deep neural network models on analyzing tabular-style datasets that lack strong multi-scale temporal or spatial structures. Low-bias tree-based models can also be more accurate and interpretable than simple high-bias models (i.e., linear regression models) as the degree of non-linearity in the data increases [1, 2]. However, complex tree-based models like RF are generally “black-box”, despite having built-in but highly compressed summary of global feature importance (model-level) [1, 3-5]. Their limited interpretability on directional magnitudes and local explanations (single-observation level) may hinder their acceptance in high-stakes decision-making domains like healthcare, where the patterns uncovered by a model are often more crucial than its predictive performance.

Post-hoc explanation methods like SHAP (SHapely Additive exPlanations) have been increasingly used to provide interpretability for complex models [6]. Having a solid theoretical foundation in game theory, SHAP values are based on the Shapley values [7] that quantify the marginal contribution of each feature (player) in a model (game) to the prediction (payout) [6]. TreeExplainer, in particular, provides faster local explanations with theoretical guaranteed consistency (monotonicity), strong performance, and explanations agreeable with human intuition for tree-based models [1]. In SHAP, the feature effect is a measure of how much the value of a specific feature influences the prediction made by the model. By visualizing numerous local explanations (individual cases), we can better understand the attributions of key features on a global level (overall model) while retaining local faithfulness to the original model. Van den Bosch and colleagues [8] used regression coefficients and SHAP values

analyses to identify risk factors associated with 30-day mortality among patients undergoing colorectal cancer surgery. Gong and colleagues [9] also developed a ML framework for acute kidney injury prediction and interpretation, using SHAP values to assess feature contributions and identify specific patient impacts.

References:

1. Lundberg SM, Erion G, Chen H, DeGrave A, Prutkin JM, Nair B, et al. From local explanations to global understanding with explainable AI for trees. Nat Mach Intell. Jan 2020;2(1):56-67. [doi: 10.1038/s42256-019-0138-9] [Medline: 32607472]

2. Ariza-Garzon MJ, Arroyo J, Caparrini A, Segovia-Vargas MJ. Explainability of a machine learning granting scoring model in peer-to-peer lending. IEEE Access. 2020;8:64873-64890. [doi: 10.1109/access.2020.2984412]

3. Murdoch WJ, Singh C, Kumbier K, Abbasi-Asl R, Yu B. Definitions, methods, and applications in interpretable machine learning. Proc Natl Acad Sci U S A. Oct 16, 2019;116(44):22071-22080. [doi: 10.1073/pnas.1900654116]

4. Molnar C. Interpretable Machine Learning: A Guide for Making Black Box Models Explainable. Victoria, CA. Leanpub; 2020. URL: https://christophm.github.io/interpretable-ml-book/ [accessed 2021-05-22]

5. Thorsen-Meyer HC, Nielsen AB, Nielsen AP, Kaas-Hansen BS, Toft P, Schierbeck J, et al. Dynamic and explainable machine learning prediction of mortality in patients in the intensive care unit: a retrospective study of high-frequency data in electronic patient records. Lancet Digit Health. Apr 2020;2(4):e179-e191. [doi: 10.1016/s2589-7500(20)30018-2]

6. Lundberg SM, Lee SI. A unified approach to interpreting model predictions. In: Proceedings of the 31st International Conference on Neural Information Processing Systems. 2017. Presented at: NIPS'17; December 4-9, 2017; Long Beach, CA.

7. Shapley L. A value for n-person games. In: Kuhn H, Tucker A, editors. Contributions to the Theory of Games II. Princeton, NJ. Princeton University Press; 1953;307-317.

8. van den Bosch T, Warps AL, de Nerée tot Babberich MP, Stamm C, Geerts BF, Vermeulen L, et al. Predictors of 30-day mortality among Dutch patients undergoing colorectal cancer surgery, 2011-2016. JAMA Netw Open. Apr 26, 2021;4(4):e217737. [doi: 10.1001/jamanetworkopen.2021.7737]

9. Gong K, Lee HK, Yu K, Xie X, Li J. A prediction and interpretation framework of acute kidney injury in critical care. J Biomed Inform. Jan 2021;113:103653. [FREE Full text] [doi: 10.1016/j.jbi.2020.103653]
